# **Understanding Mechanisms that Maintain Social Anxiety Disorder in Autistic Individuals Through the **Clark and Wells (1995)** Model and Beyond: A Systematic Review**

**DOI:** 10.1007/s10567-024-00509-z

**Published:** 2024-11-19

**Authors:** Jiedi Lei, Charlotte Mason, Ailsa Russell, Matthew J. Hollocks, Eleanor Leigh

**Affiliations:** 1 Department of Psychiatry, University of Oxford, Oxford, UK; 2 Institute of Psychiatry, Psychology & Neuroscience, King’s College London, London, UK; 3 Department of Experimental Psychology, University of Oxford, Oxford, UK; 4 Centre for Applied Autism Research, Department of Psychology, University of Bath, Bath, UK

**Keywords:** Social anxiety, Autism spectrum disorder, Clark and wells, Cognitive behaviour therapy, Cognitive model

## Abstract

Given the high co-occurrence of social anxiety in autism, no reviews to date have explored how cognitive and behavioural mechanisms identified to maintain social anxiety in non-autistic individuals may operate in autistic individuals. This systematic review evaluated: (1) empirical evidence underlying the Clark and Wells (1995) Cognitive Model of Social Anxiety in autistic individuals; (2) how vulnerability factors identified from autism literature (beyond core autistic traits) may be associated with social anxiety beyond the cognitive model. Published peer-reviewed English articles until 27th November 2023 were retrieved from PubMed, EMBASE, Ovid MEDLINE and PsycINFO. Quality appraisal and risk of bias were assessed using The Standard Quality Assessment Criteria for Evaluating Primary Research papers from a Variety of Fields tool. 47 articles met full inclusion criteria and included autistic individuals (with clinical diagnosis), a measure of social anxiety, and a mechanism outlined by either (or both) research questions. The majority of the 3340 participants across studies were male without intellectual disability, White/Caucasian, with 7 studies reporting an average age above 30 years old. Most studies used only self-report measures that may have inflated associations observed between social anxiety and mechanisms. All studies employed cross-sectional design, and no causality inferences could be drawn. Methodological issues around potential construct overlaps between social anxiety and autistic traits are highlighted. Overall, there is evidence in support of the as reported by Clark and Well (in: Heimberg (eds), A cognitive model of social phobia, The Guilford Press, 1995) model, as greater fear of negative evaluation from others, use of safety behaviours and somatic symptoms, and peer victimisation were associated with greater social anxiety. The review contributes evidence in support of autism specific contextual, predisposing/antecedent and maintenance factors of social anxiety beyond the original Clark and Well (in: Heimberg (eds), A cognitive model of social phobia, The Guilford Press, 1995) model. Given the potential for considerable heterogeneity for each highlighted process at an individual level across autistic individuals, clinicians can broaden formulation conversations with autistic clients to understand how autism related factors may influence the development and maintenance of social anxiety symptoms when applying and adapting the Clark and Well (in: Heimberg (eds), A cognitive model of social phobia, The Guilford Press, 1995) model.

**Supplementary Information:**

The online version contains supplementary material available at 10.1007/s10567-024-00509-z.

## Introduction

Autism Spectrum Disorder (hereafter autism) is a neurodevelopmental condition characterised by social communication differences, restricted and repetitive patterns of behaviours and interests, and sensory differences (American Psychiatric Association, 2013). Many autistic individuals experience anxiety in social situations, and Social Anxiety Disorder (SAD) is characterised by a persistent fear of being scrutinised or negatively evaluated by other people in social situations, that leads to persistent anxiety over time (American Psychiatric Association, 2013). One meta-analysis found that up to 29% of autistic adults have current co-occurrence of social anxiety symptoms, and a lifetime prevalence rate of 20% for SAD (Hollocks et al., 2019). The co-occurrence rate is even higher across development, with between 29 and 57% of autistic children and adolescents experiencing co-occurring SAD (Bellini, 2006; Hollocks et al., 2022; Kuusikko et al., 2008; Simonoff et al., 2008), a rate that is significantly higher than the 7–13% cited in non-autistic adolescent literature (Kessler et al., 2005). Left untreated, social anxiety (SA) can have many long-term negative consequences on individuals’ quality of life, ranging from poor quality interpersonal relationships, poor academic and employment achievements, and greater dissatisfaction in daily life (Leigh & Clark, 2018; Stein & Kean, 2000).

A number of cognitive and behavioural models have been put forward to explain the persistence of SA (e.g. Clark & Wells, 1995; Rapee & Heimberg, 1997) which share many common maintenance processes including negative social-evaluative cognitions including fear of negative evaluation from others, self-focussed attention, and a range of safety behaviours that include avoidance as well as wanting to escape the social situation (a detailed comparison across different social anxiety models have been summarised in a systematic review by Wong & Rapee, 2016). There has been a number of important efforts to develop interventions for social anxiety specifically in children and young people, a summary is provided by Mesa et al. (2015) which include a number of social skill based treatment programmes that largely focus on skill-building and use of exposure tasks to elicit changes in beliefs about oneself, social situations and other people, and also reduce social avoidance (Detweiler et al., 2014). In this systematic review, we choose to focus on Clark and Wells (1995) model as it is one of the two theoretical models (the other being the Heimberg model (Rapee & Heimberg, 1997) underpinning first-line of treatment for adults and recommended for children and young people by the Guideline Development Group (National Institute for Health & Care Excellence, 2013), yet no studies to date have evaluated the extent to which empirical evidence support the extension and adaptation of this model to address co-occurring social anxiety amongst autistic individuals across the lifespan.

According to Clark and Wells (1995), socially anxious individuals believe they will come across poorly in social situations and be judged or rejected by others [negative social-evaluative cognitions].^1^ As a result, they tend to interpret ambiguous social situations in a negative manner and catastrophise mildly negative social experiences. According to the model, social anxiety is maintained partly by focusing their attention internally to monitor self-presentation [self-focus] and increased awareness of anxiety related bodily sensations. Self-focussed attention inadvertently reduces one’s ability to notice social cues in the external environment that may contradict one’s negative interpretations of the social event. Instead, individuals attend to internal feelings of anxiety and overly negative impressions or images to infer how they appear to others. Individuals may then engage in a range of avoidance and impression management behaviours to try and prevent feared outcomes from occurring (i.e. to keep themselves safe in the social situation) [avoidance behaviour, safety behaviours, escape behaviour]. However, safety behaviours serve to maintain social anxiety over time as they increase self-focussed attention, prevent the individual from learning that the feared outcome may not have occurred in the absence of safety behaviours, negatively affect the social interaction, and may even cause the feared outcomes to occur or draw further attention to oneself (e.g. holding a glass tightly to prevent spilling a drink might increase the chance of spillage) [performance deficits due to anxiety]. Further mechanisms that maintain anxiety overtime include anticipatory worry prior to social situations that may result in behaviours such as mental rehearsal, as well as post-event rumination after a social situation [anticipatory processing and post-event processing] (Clark & Wells, 1995).

Cognitive therapy for SAD (CT-SAD) is designed to specifically target and reverse the mechanisms outlined in the Clark and Wells (1995) model. Using randomised controlled trials, CT-SAD has been shown to have superior therapeutic effects compared to a range of other medication-based (Clark et al., 2003; Mörtberg et al., 2007) and psychological therapies (Clark et al., 2006; Stangier et al., 2003, 2011) when working with neurotypical adults (Mayo-Wilson et al., 2014), and more recently with neurotypical adolescents (Ingul et al., 2014; Leigh & Clark, 2023). However, the evidence behind extending and adapting CT-SAD for autistic individuals remains scarce. Autistic people have differences in social communication and neurocognition that may mean evidence-based interventions are less accessible to them. There is a growing evidence base for the effectiveness of cognitive behavioural interventions for emotional disorders if adapted to meet autistic people’s needs (e.g. J. J. Wood et al., 2020). However, there is a lack of focussed evidence about adapting NICE recommended CBT for SA. One systematic review by Spain and colleagues (2017) found only four single patient case studies all with autistic males (aged 6–47 years) looked at changes in SA symptoms following CBT for social anxiety (Cardaciotto & Herbert, 2004; Paul Wright, 2013; Schleismann & Gillis, 2011; Turner & Hammond, 2016). The review noted that common intervention techniques included psychoeducation and social skills interventions that are not part of standard individual CBT for SA as recommended by NICE guidelines. Studies used more anxiety hierarchy and exposure-based tasks as well as cognitive restructuring and positive self-talk to support autistic individuals with SA, which resemble group based CBT for SA (Albano et al., 1995). However, none of the studies explicitly tested how specific cognitive and behavioural mechanisms such as those outlined in the Clark and Wells (1995) model of SA may influence treatment efficacy (Spain et al., 2017).

Reviews of interventions in autism have begun to highlight the importance of understanding key mechanisms of change underlying psychosocial interventions for mental health difficulties in autistic individuals (Lerner et al., 2012), so that clinicians can better understand the more nuanced questions of “*why and how does it work, for whom, under what conditions and when”* (Kazdin, 2007; Lerner et al., 2012; Norcross & Wampold, 2011). To date, no systematic reviews have been completed to examine the cognitive and behavioural maintenance mechanisms of SA in autistic individuals, including those invoked in theoretical models such as that of Clark and Wells (1995), or other processes that have been hypothesised to play a role in maintenance of anxiety in autistic individuals. Beyond social skills differences that may have negative consequences on peer interactions (Bellini, 2006), other factors to include during formulation that may tap into mechanisms of change underlying psychosocial interventions include autism social identity and solidarity (i.e. feeling a sense of connection with the autistic community) (Cooper et al., 2022a, 2022b; Hedley & Young, 2006), cognitive flexibility (Lei et al., 2022; Ozsivadjian et al., 2021), interoceptive awareness (i.e. recognition of one’s internal bodily states), alexithymia (i.e. difficulties in identifying one’s emotions) (Pickard et al., 2020), and social camouflaging via hiding one’s autism traits and trying to compensate for social communication differences to fit in with the neurotypical world (Hull et al., 2021).

Critically evaluating the state of evidence to understand the associations between SA and mechanisms proposed in the Clark and Wells (1995) model, and other maintenance factors of anxiety in autism offers valuable insight about the empirical basis for evidence-based interventions. Given that previous systematic reviews identified few studies that examined SA interventions in autism (Spain et al., 2017, 2018), we also adopted a lifespan perspective in our review to examine potential mechanisms underlying the maintenance of SA amongst autistic individuals. However, given that there may be key developmental differences in the aetiology and maintenance of SA from pre-adolescence to adolescence and adulthood (Halldorsson & Creswell, 2017), we are interested in exploring how significant associations between key cognitive and behavioural maintenance factors and SA symptom severity may change across development.

To extend our knowledge beyond understanding the relationship between SA and autism symptom severity (Spain et al., 2018) by adopting a more mechanistic approach towards understanding the presentation of SA in autism, the current systematic review has two main research questions (RQ) as stated below:

1. What is the current state of research evidence that examine constructs within the cognitive model of Clark and Wells (1995) for SA in autistic individuals?
2. Which additional mechanisms have been evaluated in autistic individuals that can inform formulation alongside Clark and Wells (1995) model for understanding SA in autistic individuals?

Through a closer examination of the quantity and quality of research evidence supporting maintenance factors of SA outlined in the Clark and Wells (1995) model, we hope to elucidate research gaps, future directions, and additional factors to consider when applying current models of SA in autistic individuals.

## Methods

### Search Strategy

This systematic review followed the PRISMA 2020 Checklist (see Appendix A, Page et al., 2021) with a registered protocol on Prospero (CRD42023437166). Peer-reviewed articles published in English until 27th November 2023 were retrieved from PubMed, EMBASE, Ovid MEDLINE and PsycINFO. The earliest article retrieved was published in 1957. We completed searches using different key words for each of the two research questions. Both searches contained synonyms of keywords including SA and autism. Additional search terms for RQ 1 included maintenance factors identified in the Clark and Wells (1995) cognitive model of social anxiety, and for RQ 2 included additional processes identified in autism literature associated with SA (See Appendix B for full search strategy). Results were collated using Excel, and after removing duplicates, titles and abstracts were first screened, and full-text articles were reviewed based on inclusion/exclusion criteria. Reference lists of all included studies were screened to identify potentially relevant articles missing from the initial search.

### Study Selection

**Fig. 1:**
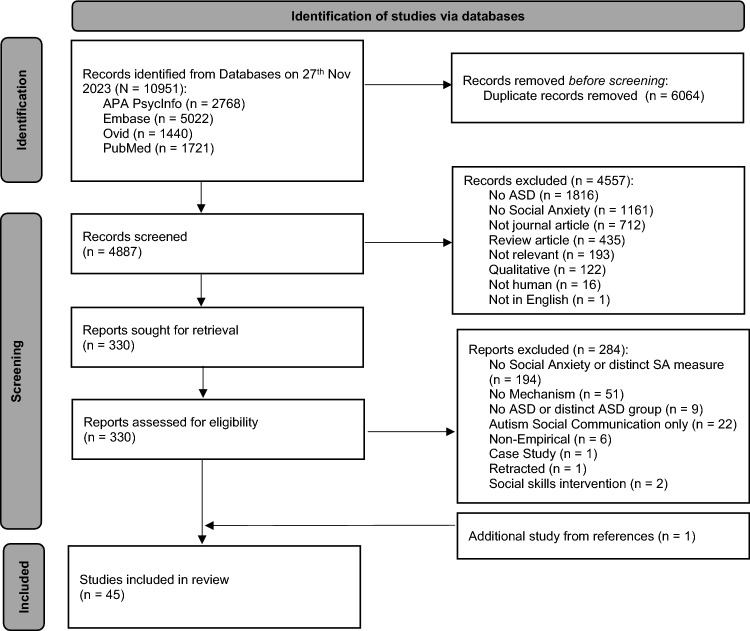
PRISMA flowchart showing identification and selection of articles included in the systematic review

Table 1 shows the detailed inclusion/exclusion criteria following the Participant, Exposure, Comparison, Outcome (PECO) framework (Dekkers et al., 2019). Cross-sectional and longitudinal quantitative studies published in English in peer-reviewed journals were included for review. Qualitative studies, opinion articles, reviews/meta-analyses, grey literature, and non-English publications were excluded from the review. The first author independently screened all studies at each stage, and the second author independently completed title/abstract screening (*n* = 485, *κ* = 0.66, 95% CI [0.56 to 0.75]) and full-text screening (*n* = 32, *κ* = 1) for 10% of randomly selected studies, indicating moderate to almost perfect interrater reliability (J. Cohen, 1960; McHugh, 2012). All discrepancies were discussed by referring to the inclusion/exclusion criteria and in consultation with authors with expertise on the topic, until a consensus was reached. Figure 1 shows the PRISMA flowchart of articles screened and selected at each stage.

**Table 1:** Summary of inclusion and exclusion criteria as per Participant Exposure Comparison Outcome (PECO) framework

Inclusion criteria	Exclusion criteria
***Participant***	
• Have a clinical diagnosis of Autism Spectrum Disorder or equivalent (e.g. childhood autism (ICD-10)/Autistic Disorder (DSM-IV), Asperger’s Syndrome, Pervasive Developmental Disorder – Not Otherwise Specified) • Clinical diagnosis should be provided (ideally but not exclusively) by a healthcare or educational professional via clinical assessment measures	• Does not include individuals with clinical diagnosis of Autism Spectrum Disorder or equivalent
***Exposure***	
• Include at least one instrument to measure social anxiety symptom severity, but not limited to the tests and measures stated below, which have been identified by a systematic review by Leigh and Clark (2018) and Spain et al. (2018) ADIS-IV = Anxiety Disorders Interview Schedule for DSM-IV BFNE = Brief Fear of Negative Evaluation Scale CAPA = Child and Adolescent Psychiatric Assessment CASI = Child and Adolescent Symptom Inventory K-SADS-PL = Schedule for Affective Disorder sand Schizophrenia for School Aged Children, present and lifetime version LSAS = Liebowitz Social Anxiety Scale MASC = Multidimensional Anxiety Scale for Children MINI = Mini Interpersonal Neuropsychiatric Interview RCADS = Revised Children’s Anxiety and Depression Scale SAS (-A; -CR) = Social Anxiety Scale (for Adolescents, for Children-Revised) SASPA = Social Anxiety Scale for People With ASD SCARED = Screen for Child Anxiety Related Emotional Disorder SCAS = Spence Children’s Anxiety Scale SCID = Structured Clinical Interview for DSM-IV SDS = Social Desirability Scale SIAS = Social Interaction Anxiety Scale SPAI (-C) = Social Phobia Anxiety Inventory (for Children) SPIN = Social Phobia Inventory SPS = Social Phobia Scale SSRS = Social Skills Rating Scale SWQ = Social Worries Questionnaire	• Does not include any instrument to measure social anxiety
***Comparison (optional)***	
• May include age-matched sample of neurotypical or non-autistic individuals with or without social anxiety symptoms as a comparison group. If the study meets the requirement under Participant and Exposure of the PECO criteria, absence of a comparison group will not lead to the exclusion of the study in the systematic review, as a comparison group is optional and not required to address the stated research question	• Not applicable
***Outcome***	
For Research Question 1: • Must meet the inclusion criteria for Participant and Exposure, and include at least one measure of at least one psychological variable identified in the cognitive model of social anxiety by Clark and Wells (1995). A list of psychological variables is found in Clark and Wells (1995) and in the systematic review by Leigh and Clark (2018): 1) Negative social attitudes and cognitions 2) Negative interpretation bias 3) Self-focussed attention 4) Reduced processing of external social cues 5) Negative observer-perspective social images 6) Use of internal information 7) Use of safety behaviours 8) Pre- and post-event processing For Research Question 2: • Must meet the inclusion criteria for Participant and Exposure and may include at least one measure of the following processes that have shown associations with social anxiety symptom severity amongst autistic individuals: 1) Social skills, knowledge, motivation and competence beyond autism symptom severity (Lerner et al., 2012) 2) Social camouflaging / masking (Hull et al., 2021) 3) Autism identity and self-comparison (Cooper et al., 2022a, 2022b; Hedley & Young, 2006) 4) Cognitive flexibility (Lei et al., 2022; Ozsivadjian et al., 2021) 5) Interoceptive awareness and self-regulation (Pickard et al., 2020) 6) Alexithymia (Pickard et al., 2020)	• Does not include any measure of psychological variables identified in Clark and Wells (1995) model of social anxiety OR any of the mechanisms outlined under research question 2 that include more autism specific factors to consider

### Quality Appraisal

The first and second author used The Standard Quality Assessment Criteria for Evaluating Primary Research papers from a Variety of Fields (Kmet et al., 2004) to complete quality appraisal of all included studies (available upon request from authors). The recommended cut-off threshold for inclusion following quality appraisal ranges from stringent (0.75) to more liberal (0.55) (Kmet et al., 2004), and the lowest quality appraisal score of studies included in this review is 0.61. The first and second author completed quality appraisals for all studies. All disagreements were discussed by referring to the Quality Appraisal tool until consensus was reached.

### Data Extraction

We extracted the following information for each included study: (1) author, year, and country of publication; (2) sample size (n, % male) and recruitment channel; (3) autism diagnosis (criteria or measure used, and autism symptom severity); (4) age, IQ, ethnicity (% Caucasian), and co-occurring conditions; (5) SA measure; (6) mechanism measure; (7) interaction between mechanism and SA. A summary of all studies that explored maintenance factors in the original Clark and Wells (1995) model is shown in Table 2, additional vulnerability factors associated with SA in autism are summarised in Tables 3 and 4.

**Table 2:** Study characteristics of autistic participants included in the 45 full-text articles

	**Aim 1 – Clark and Well model (n = 901; 13 studies; 13 independent samples)**		**Aim 2 – Autism related factors (n = 2248; 37 studies; 32 independent samples)**	
	**M (SD)**	**Range**	**M (SD)**	**Range**
**Sample size**	69.31 (50.92)	13—192	70.25 (73.21)	8—354
**% Male**	69.53 (21.89)	28 – 100	74.20 (20.61)	27.87—100
**Age (Years)**	20.17 (9.84)	11.91 – 49.05	19.96 (11.34)	8.44 – 49.05
**FSIQ**	(7 studies)		(21 studies, 19 independent samples)	
	104.99 (6.33)	98.16 – 108.38	102.65 (15.65)	45 – 118.65
**Ethnicity**	(%—4 studies)		(%—9 studies)	
Caucasian	81.06 (3.72)	77.78 – 85.94	76.61 (17.39)	36.67 – 90.08
Mixed/Other ethnicity	12.07 (1.25)	10.71 – 13.58	5.97 (9.98)	0 – 30
Black	2.16 (2.54)	0 – 4.92	3.84 (5.74)	0 – 17.39
Asian	4.71 (4.30)	1.56 – 10.71	14.69 (8.63)	4.96 – 33.33
**Study quality**	0.82 (0.09)	0.64 – 0.91	0.92 (0.03)	0.61 – 0.95
**Recruitment**	(*n* = studies)		(n = studies, samples in brackets)	
Clinical sites (hospitals / clinic)	7		23 (19 samples)	
Community settings	5		16 (15 samples)	
School/ University	8		10 (8 samples)	
Online	2		4 (4 samples)	
Longitudinal/cohort datasets	0		1 (1 sample)	
**Comorbidities**	(*n* = participants; 6 studies)		(n = participants; 12 studies; 10 independent samples)	
ADHD	51		74	
Depression	21		202	
Anxiety (unspecified)	5		9	
GAD	44		272	
SAD	122		148	
Simple phobia	2		2	
Separation Anxiety	14		49	
Panic	22		36	
PTSD	2		2	
OCD	32		62	
Eating Disorder	6		6	
Learning/Language related diagnosis (including dyslexia, dyspraxia)	37		41	
Selective Mutism	-		38	
**Social Anxiety Measure**	(*n* = studies)		(n = studies)	
Self-report	13		26	
Caregiver report	2		14	
Clinician/Teacher report	1		4	
**Mechanism Measure**	(*n* = studies)		(n = studies)	
Self-report	10		14	
Caregiver report	4		16	
Clinician/Teacher measure	2		4	
Task-based performance measure	2		13	

*ADHD* attention deficit hyperactive disorder, *FSIQ* full scale IQ, *GAD* generalised anxiety disorder, *OCD* obsessive compulsive disorder, *PTSD* post-traumatic stress disorder, *SAD* social anxiety disorder

**Table 3:** Summary of studies exploring factors in the Clark and Wells (1995) cognitive model of social anxiety

**Authors** (Year, Country)	**Sample Size** (N, male) **Recruitment Channel**	**Diagnosis** (Criteria/ Measure)	**Age** (M; SD) **IQ** (M; SD) **Ethnicity** (Caucasian N; %) **Co-occurring conditions in ASD group** (N; %)	**Social Anxiety Measure**	**Mechanism Measure**	**Outcome**	**Quality Score**
A. ***Negative Social Cognitions (n* = *3)***							
Lei et al. (2023); UK	ASD: 61 (17) NT: 54 (11) Clinic; School/University; Online	Clinical diagnosis *AutSev* (ASD): AQ-28 77.93 (10.23)	***Age:*** ASD: 16.34 (1.69)***;*** NT: 16.02 (1.56) ***IQ:*** none reported ***Ethnicity:*** ASD: 50 (81.97)***;*** NT: 37 (68.52) ***Co-occurring:*** 9 ADHD, 22 GAD, 19 SAD, 21 OCD, 1 Panic, 2 PTSD, 17 Depression, 6 Eating disorder	SPIN (self)	ASCQ (self)	***Covariates controlled for:*** GAD and depression symptom severity, groups matched on SA ***Social anxiety:*** ASD and Non-ASD matched ***Social cognitions:*** ASD and Non-ASD matched ***Interaction:*** ASD: ↑ SA = ↑ Social anxiety related social cognitions (*r* = .69, *p* < .001)	0.91
Wilson et al. (2023); UK	ASD: 192 (56) Non-ASD: 69 (26) Self-identify ASD: 51 (14) Broad Aut Phenotype (BAP): 24 (9) Community; Online	Clinical diagnosis; AQ *AutSev* (ASD): AQ-10 7.89 (1.79)	***Age:*** ASD: 40.95 (13.62); N-ASD: 40.30 (17.28); S-ASD: 41.98 (13.95); BAP: 32.82 (11.16) ***IQ:*** none reported ***Ethnicity:*** ASD: 165 (86); N-ASD: 61 (88); S-ASD: 42 (82); BAP: 17 (71) ***Co-occurring:*** 58 SAD, 36 ADHD, 13 dyslexia, 15 dyspraxia, 9 language/learning-related diagnosis	LSAS (self)	SCogQ (self)	***Covariates controlled for:*** none reported; no association between outcome variables and age/gender ***Social anxiety*:** ASD > non-ASD (Cohen’s *d* = 1.01) ***Social cognitions:*** ASD > non-ASD (Cohen’s *d* = 0.8) ***Interaction:*** ASD: ↑ SA = ↑ Social anxiety related social cognitions (*r* = .56, *p* < .05)	0.95
Hollocks et al. (2016); UK	ASD: 21 (21) ASDAnx: 34 (34) Non-ASD: 28 (28) Clinical site	Clinical diagnosis, ADI-R/ADOS, SCQ *AutSev:* SCQ 19.4 (5.7) for ASD, 24.7 (5.8) for ASDAnx	***Age:*** ASD: 13 (1.9); ASDAnx: 12.7 (1.9); Non-ASD: 13.9 (1.8) ***IQ:*** ASD: 103 (16.7); ASDAnx: 99.7 (10.9); Non-ASD: 116 (9.5) ***Ethnicity:*** none reported ***Co-occurring:*** 62% ≥ 1 anxiety; 18% ≥ 3 anxiety; 21 panic/agoraphobia, 22 GAD, 14 separation, 2 simple phobia, 4 SAD, 11 OCD	SCAS-P (caregiver) SCAS-C (self)	Interpretation bias: Ambiguous Situation Interview (self)	***Covariates controlled for****:* IQ ***Social anxiety****:* ASDAnx > ASD > non-ASD ***Interpretation bias****:* ASDAnx > non-ASD on negative interpretations for social and non-social situations, no group differences in physical threat situations ***Interaction****:* SA rated by parent and child not associated with total interpretation bias (*r* = .05 to .17, *p* > .05), social interpretation bias (*r* = .1 to .16, *p* > .05), or physical interpretation bias (*r* = -.11 to .28, *p* > .05)	0.86
B. ***Perceived Social Danger (including fear of negative evaluation from other) (n* = *4)***							
Boulton et al. (2021); Australia	ASD: 102 (73) SAD: 316 (178) ASD + SAD: 60 (40) Clinical site	Clinical diagnosis; ADOS (DSM-5); ADIS DSM-IV (for SAD) *AutSev:* ADOS-2: 9.59 (2.79) for ASD group; 9.08 (2.48) for ASD + SAD group	***Age:*** ASD: 23.23 (6.61); SAD: 24.64 (7.11); ASD + SAD: 23.57 (3.29) ***IQ:*** ASD: 108.38 (7.22); SAD: 111.21 (3.35); ASD + SAD: 107.58 (6.40) ***Ethnicity:*** none reported ***Co-occurring:*** SAD	LSAS-SR (self) SIAS (self) SPS (self)	BFNE (self)	***Covariates controlled for:*** IQ ***Social anxiety*:** n above clinical cut-off for each measure: ASD: 67/102 on LSAS-SR; 73/102 on SIAS; 57/102 on SPS SAD: 227/316 on LSAS-SR; 263/316 on SIAS; 210/316 on SPS ***Interaction:*** ASD group: ↑ FNE = ↑ SA (*r* = 0.64, *p* < .001); fear (*r* = .70, *p* < .001), ↑ avoidance (*r* = .52, *p* < .001); ↑ social interaction anxiety (*r* = 0.67, *p* < .0017) ↑ being scrutinised by others (*r* = 0.62, *p* < .001) SA group: ↑ FNE = ↑ SA (*r* = 0.61, *p* < .001); fear (*r* = .66, *p* < .001) ↑ avoidance (*r* = .54, *p* < .001); ↑ social interaction anxiety (*r* = .69, *p* < .001) ↑ being scrutinised by others (*r* = 0.61, *p* < .001)	0.82
Gaziel-Guttman et al. (2023); Israel	ASD: 33 (28) Non-ASD: 38 (32) Community	Clinical diagnosis by DSM-IV-TR or DSM-5; ADOS *AutSev:* AQ 30.7 (3.89)	***Age:*** ASD: 23.55 (2.37); Non-ASD: 24.29 (2.18) ***IQ:*** ASD: 116.7 (6.94); Non-ASD: 118.58 (6.52) ***Ethnicity:*** none reported ***Co-occurring:*** 6 ADHD, 5 anxiety, 4 depression	LSAS (self)	GASP (self)	***Covariates controlled for:*** none reported; no group diff in gender, age, IQ ***Social Anxiety:*** ASD > non-ASD on total, fear, avoidance ***Shame:*** ASD < non-ASD on total and negative-self-evaluation subscale; no group diff on withdraw subscale ***Interaction:*** • ASD: ↑ Avoidance = ↓ Negative self-evaluation (*r* = -.39, *p* < .05) ASD vs. Non-ASD: ↑ Shame = ↑ SA only in non-ASD group (fear subscale: *r* = .53, *p* < .001; withdraw subscale: *r* = .42, *p* < .001); ↑ Shame = ↑ Withdraw in both groups (ASD: *r* = .85, *p* < .001; non-ASD: *r* = .89, *p* < .001)	0.91
Maddox et al. (2015); USA	ASD: 28 (15) SAD: 26 (13) Non-Clinical: 25 (12) University	Clinical diagnosis, ADOS-2 and ADI-R *AutSev*: SRS-2-A 68.43 (9.82)	***Age:*** ASD: 23.93 (6.92); SAD: 25.96 (7.12); NC: 24.78 (7.31) ***IQ:*** ASD: 106.68 (16.58); SAD: 108.85 (10.51); NC: 114.24. (10.78) ***Ethnicity:*** ASD: 22 (78.6); SAD: 20 (76.9)***;*** NC: 17 (68) ***Co-occurring:*** SAD (14, 50%)	ADIS-IV: SAD module SASPA (clinician) SIAS (self)	BFNE (self)	***Covariates controlled for:*** no between group diff in gender, age and IQ not associated with social anxiety ***Social anxiety:*** 14 (50%) of ASD group > SAD cut-off; Most highly endorsed items on ADIS-IV: ASD + SAD (n = 14): 1) attending parties; 2) formal speaking; 3) speaking with unfamiliar people SAD: 1) formal speaking; 2) being assertive to ask others to change behaviours; 3) speaking with unfamiliar people ***Interaction:*** • ASD + SAD > SAD on social interaction anxiety – especially on “making eye contact”; “talk about myself or my feelings”, “mix comfortably with peers”, “making friends of my own age”, “talking with others” • ASD + SAD vs. SAD: ↑ SA during middle childhood • 50% of ASD + SAD and 8% of SAD group attributed autism related social skills impairment to the development and maintenance of SA	0.86
Pickard et al. (2020); UK	ASD: 61 (42) NT: 62 (26) School	Clinical and school diagnosis; SCQ-L and SRS-2 *AutSev* (ASD): SRS-2 77.47 (10.65); SCQ lifetime 20.94 (6.98)	***Age:*** ASD: 13.46 (1.77); NT: 13.52 (1.57) ***IQ:*** ASD: 98.16 (13.99)***;*** NT: 100.76 (11.55) ***Ethnicity****:* none reported ***Co-occurring:*** none reported	LSAS (self)	BFNE (self)	***Covariates controlled for:*** sex ***Social anxiety:*** 52.4% of ASD vs. 54.8% NT > SA cut-of ***FNE:*** no difference between NT and ASD (*d* = .22, *p* > .05) ***Interaction:*** • ↑ FNE = ↑ SA in ASD (*r* = .72, *p* < .001) and NT (*r* = .66, *p* < .001) • ↑ FNE = ↑ autistic traits in NT (*r* = .31, *p* < .05) and not in ASD (*r* = .18,* p* > .05)	0.91
C. ***Processing of Self as Social Object (n* = *2)***							
Wilson et al. (2023); UK	ASD: 192 (56) Non-ASD: 69 (26) Self-identify ASD: 51 (14) Broad Aut Phenotype (BAP): 24 (9) Community Online	Clinical diagnosis; AQ *AutSev* (ASD): AQ-10 7.89 (1.79)	***Age:*** ASD: 40.95 (13.62); N-ASD: 40.30 (17.28); S-ASD: 41.98 (13.95); BAP: 32.82 (11.16) ***IQ:*** none reported ***Ethnicity:*** ASD: 165 (86); N-ASD: 61 (88); S-ASD: 42 (82); BAP: 17 (71) ***Co-occurring:*** 58 SAD, 36 ADHD, 13 dyslexia, 15 dyspraxia, 9 language/learning-related diagnosis	LSAS (self)	FAQ (self)	***Covariates controlled for:*** none reported; no association between outcome variable sand age/gender ***Social anxiety*:** ASD > non-ASD (Cohen’s *d* = .1.01) ***Self-focussed attention:*** ASD > non-ASD (Cohen’s *d* = 0.6) ***Interaction:*** ASD: Self-focussed attention not associated with SA (*r* = 0.41, *p* > .05)	0.95
Wood et al. (2022); UK	Study 1: High SA: 41 (29) Low SA: 30 (26) Study 2: 76 (44) Community; School/University	Clinical diagnosis *AutSev:* none reported	Study 1: ***Age:*** High SA: 18.17 (2.46); Low SA: 17.33 (1.42) ***IQ:*** none reported ***Ethnicity:*** none reported Study 2: ***Age:*** 17.91 (1.93) ***IQ:*** none reported ***Ethnicity:*** none reported ***Co-occurring:*** none reported for either	SAS-A (self) SPIN (self)	FAQ (self) SCS (self) Self-imagery: Performance scale	***Covariates controlled for:*** none reported. No between group diff on demographics ***Social anxiety:*** High SA > Low SA on FNE, anxiety in general and new situations ***Social performance:*** High SA < Low SA on self- and observer-rated social performance ***Interaction:*** • ↑ SA = ↓ Self-rated performance (*r* = -.415, *p* < .001) (fully mediated by greater interoceptive sensibility; partially mediated by public social consciousness/trait SFA) • ↑ SA = ↑ Self-focussed attention (*r* = .332, *p* = .003)	0.82
***D. Physical arousal and interoceptive sensibility (use of internal information, n* = *3)***							
Bellini et al. (2006)	ASD: 41 (35) School	Clinical diagnosis *AutSev:* none reported	***Age:*** 14.22 ***FSIQ:*** 99.94 (18.81) ***Ethnicity:*** none reported ***Co-occurring:*** none reported	SAS-A (self)	MASC-Physical Symptoms Scale (self)	***Covariates controlled for:*** none reported ***Interaction:*** ↑ physical arousal = ↑ SA (*β* = .52, *p* < .01)	0.64
Pickard et al. (2020); UK	ASD: 61 (42) NT: 62 (26) School	Clinical and school diagnosis *AutSev* (ASD): SRS-2 77.47 (10.65); SCQ lifetime 20.94 (6.98)	***Age:*** ASD: 13.46 (1.77); NT: 13.52 (1.57) ***IQ:*** ASD: 98.16 (13.99); NT: 100.76 (11.55) ***Ethnicity:*** none reported ***Co-occurring:*** none reported	LSAS (self)	BPQ-Awareness (self)	***Covariates controlled for:*** sex ***Social anxiety:*** 52.4% of ASD vs. 54.8% NT > SA cut-off ***Interoceptive sensibility:*** NT > ASD (*d* = .58, *p* < .01) ***Interaction:*** ASD: ↑ SA = ↑ Interoceptive sensibility (*r* = .46, *p* < .001) NT: ↑ SA = ↑ Interoceptive sensibility (*r* = .33, *p* < .009) No difference in magnitude of correlations between groups (*Z*s = -.38–1.64, *p*s > .101)	0.91
Wood et al. (2021); UK	Study 1: High SA: 41 (29) Low SA: 30 (26) Study 2: 76 (44) Community; School/University	Clinical diagnosis *AutSev:* none reported	Study 1: ***Age:*** High SA: 18.17 (2.46); Low SA: 17.33 (1.42) ***IQ:*** none reported ***Ethnicity:*** none reported Study 2: ***Age:*** 17.91 (1.93) ***IQ:*** none reported ***Ethnicity****:* none reported ***Co-occurring:*** none reported for either	SAS-A (self) SPIN (self)	APQ (self)	***Covariates controlled for:*** none reported. No between group diff on demographics ***Social anxiety:*** High SA > Low SA on FNE, anxiety in general and new situations ***Interoceptive sensibility:*** ↑ interoceptive sensibility = ↑ state self-focussed attention (*r* = .78, *p* < .001) and trait self-focussed attention (*r* = .35, *p* = .002) ***Interaction:*** ↑ SA = ↑ interceptive sensibility (*r* = .61, *p* < .001) Association between ↑ SA and ↓ self-rated performance was fully mediated by ↑ interoceptive sensibility ↑ SA endorsed ↑ interoceptive sensibility in bodily temperature, muscle tension, and heart rate	0.92
E. ***Safety Behaviours (n* = *3)***							
Lei et al. (2023); UK	ASD: 61 (17) Non-ASD: 54 (11) Clinic; School/University; Online	Clinical diagnosis *AutSev (*ASD): AQ-28 77.93 (10.23)	***Age:*** ASD: 16.34 (1.69); Non-ASD: 16.02 (1.56) ***IQ:*** none reported ***Ethnicity:*** ASD: 50 (81.97); Non-ASD: 37 (68.52) ***Co-occurring:*** 9 ADHD, 22 GAD, 19 SAD, 21 OCD, 1 Panic, 2 PTSD, 17 Depression, 6 Eating disorder	SPIN (self)	ASBQ (self)	***Covariates controlled for:*** GAD and depression symptom severity, groups matched on SA ***Social anxiety:*** ASD and Non-ASD matched ***Social behaviours:*** ASD and Non-ASD matched on avoidance and impression management ***Interaction:*** ASD: ↑ SA = ↑ Safety behaviours total (*r* = .63, *p* < .001), including ↑ avoidance (*r* = .71, *p* < .001) and ↑impression management (*r* = .44, *p* < .001)	0.91
Perry et al. (2015)	ASD: 13 (12) Non-ASD: 13 (13) Community	Clinical diagnosis, ADOS, ADI-R *AutSev:* none reported	***Age:*** ASD: 25 (1.24); NT: 24 (0.46) ***IQ:*** Not reported ***Ethnicity:*** not reported ***Co-occurring:*** none reported	LSAS (self)	Social avoidance: interpersonal distance measured by stop-distance paradigm (self)	***Covariates controlled for*:** none reported ***Social anxiety:*** ASD and non-ASD matched ***Social avoidance:*** ASD and non-ASD matched on mean preferred distance (in meters); ASD > non-ASD on variance on preferred distance ***Interaction:*** ↑ SA = ↑ Preferred interpersonal distance in ASD (*r* = .59, *p* < .05) but not in non-ASD group	0.77
Wilson et al. (2023); UK	ASD: 192 (56) Non-ASD: 69 (26) Self-identify ASD: 51 (14) Broad Aut Phenotype (BAP): 24 (9) Community; Online	Clinical diagnosis; AQ *AutSev* (ASD): AQ-10 7.89 (1.79)	***Age:*** ASD: 40.95 (13.62); N-ASD: 40.30 (17.28); S-ASD: 41.98 (13.95); BAP: 32.82 (11.16) ***IQ:*** none reported ***Ethnicity:*** ASD: 165 (86); N-ASD: 61 (88); S-ASD: 42 (82); BAP: 17 (71) ***Co-occurring:*** 58 SAD, 36 ADHD, 13 dyslexia, 15 dyspraxia, 9 language/learning-related diagnosis	LSAS (self)	SBQ (self)	***Covariates controlled for:*** none reported; no association between outcome variable sand age/gender ***Social anxiety*:** ASD > non-ASD (Cohen’s *d* = .1.01) ***Safety behaviours:*** ASD > non-ASD (Cohen’s *d* = 0.9) ***Interaction:*** ASD < non-ASD in association between safety behaviours and SA ASD: ↑ SA = ↑ Safety behaviours (*r* = .54, *p* < .05)	0.95
***F. Social Experiences (n* = *6)***							
a) ***Peer Victimisation and Social Problems (n* = *3)***							
Ambler et al. (2015); Australia	ASD: 52 (42) non-ASD: 52 (42) School	Clinical diagnosis of Asperger’s, high functioning autism, or autistic disorder without intellectual disability *AutSev:* none reported	***Age:*** ASD: 14.5 (1.77); non-ASD: 14.35 (1.68) ***IQ:*** none reported ***Ethnicity:*** none reported ***Co-occurring:*** none reported	RCMAS-2 – social anxiety scale (self)	School behaviours: SBS (teacher)	***Covariates controlled for:*** none reported; groups matched on gender and age ***Social anxiety:*** ASD > non-ASD ***Social problems:*** ASD > non-ASD ***Interaction:*** ASD: ↑ SA = ↑ social problems (*r* = .29, *p* < .05) (i.e. avoids social interaction in class, ignored/rejected by peers) Non-ASD: no significant association between SA and social problems (*r* = .17, *p* > .05)	0.77
Ung et al. (2016); USA	ASD: 81 (62) Clinical site, community	Clinical diagnosis *AutSev:* none reported	***Age:*** 11.91 (2.32) ***IQ:*** 104.1 (14.24) ***Ethnicity****:* 63 (77.8) ***Co-occurring:*** none reported	SASC-R (self)	PEQ-R (self, caregiver)	***Covariates controlled for:*** none reported ***Peer victimisation:*** In past 12 months, self-report of peer victimisation on average “a few times”, relational victimisation on average “a few times” and overt victimisation on average “a few times”. Cyberbullying reported by 9 youths as happening on average “a few times a week”. No sex differences ***Interaction:*** Child reports: ↑ peer victimisation = ↑ social avoidance (b = .13, *p* < .05), not with fear of negative evaluation (FNE; b = .03, *p* = .79) Parent reports: ↑ peer victimisation = ↑ social avoidance (b = .14, *p* < .05), not with FNE (b = .07, *p* = .14)	0.77
Van Schalkwyk et al. (2018); USA	ASD: 35 (23) Clinical site, school	Clinical diagnosis by DSM-IV or DSM-5 *AutSev:* SRS-2 total 87.3 (20.9)	***Age:*** 16.4 (1.58) ***IQ:*** none reported ***Ethnicity:*** none reported ***Co-occurring:*** none reported	MASC-2-P (caregiver) MASC-2-C (self)	Peer victimisation: report on bullying/victimisation (caregiver, self)	***Covariates controlled for:*** none reported ***Bullying:*** parent- and child-rated bullying showed non-significant negative association (*r* = -.27, *p* = .156) ***Interaction:*** ↑Parent rated bullying = ↑child-rated SA (*r* = .56, *p* = .001) and parent-rated SA (*r* = .37, *p* = .038) Child rated bullying not associated with child (*r* = -.34, *p* = .06) or parent rated (*r* = -.21, *p* = .24) SA	0.68
***b) Adverse Childhood Experiences (n* = *1)***							
Wilson et al. (2023); UK	ASD: 192 (56) Non-ASD: 69 (26) Self-identify ASD: 51 (14) Broad Aut Phenotype (BAP): 24 (9) Community; Online	Clinical diagnosis; AQ *AutSev* (ASD): AQ-10 7.89 (1.79)	***Age:*** ASD: 40.95 (13.62); N-ASD: 40.30 (17.28); S-ASD: 41.98 (13.95); BAP: 32.82 (11.16) ***IQ:*** none reported ***Ethnicity:*** ASD: 165 (86); N-ASD: 61 (88); S-ASD: 42 (82); BAP: 17 (71) ***Co-occurring:*** 58 SAD, 36 ADHD, 13 dyslexia, 15 dyspraxia, 9 language/learning-related diagnosis	LSAS (self)	VEQ-subset (self)	***Covariates controlled for:*** none reported; no association between outcome variable sand age/gender ***Social anxiety*:** ASD > non-ASD (Cohen’s *d* = .1.01) ***Interaction:*** Negative early social experiences did not explain additional variances between autism traits and greater SA; SA not significantly associated with adverse childhood social experiences (*r* = .22)	0.95

*ADHD* Attention Deficit Hyperactive Disorder, *ADI-R* Autism Diagnostic Interview-Revised, *ADOS* Autism Diagnostic Observation Schedule, *APQ* Autonomic Perception Questionnaire, *ASBQ* Adolescent Social Behaviours Questionnaire, *ASCQ* Adolescent Social Cognitions Questionnaire, *ASD* Autism Spectrum Disorder, *AQ-10* Autism-Quotient-10, *BFNE* Brief Fear of Negative Evaluation, *BPQ* Body Perception Questionnaire, *FAQ* Focus of Attention Questionnaire, *FQS* Friendship Quality Scale, *GASP* Guilt and Shame Proneness Questionnaire, *LSAS* Leibowitz Social Anxiety Scale, *OCD* Obsessive Compulsive Disorder, *MASC-2-C/P MASC* Multidimensional Anxiety Scale for Children – Child/Parent, *PEQ-R* Revised Peer Experiences Questionnaire, *RCMAS-2* Revised Children’s Manifest Anxiety Scale – 2nd Edition, *SAD* Social Anxiety disorder, *SASA* Social Anxiety Scale-Adolescents, *SASC-R* Social Anxiety Scale for Children-Revised, *SASPA* Social Anxiety Scale for People with ASD, *SBQ* Social Behaviours Questionnaire, *SBS* Student Behaviour Survey (adjustment problems; emotional distress, unusual behaviour, social problems, verbal and physical aggression, behaviour problems), *SCAS-P/C* Spence Children’s Anxiety Scale – Parent/Child, *SCS* Social Consciousness Scale, *SCogQ* Social Cognitions Questionnaire, *SIAS* Social Interaction Anxiety Scale, *SPIN* Social Phobia Inventory, *SPS* Social Phobia Scale, *SRS-2* Social Responsiveness Scale-2, *VEQ* Vulnerability Experiences Quotient

* Intervention study

**Table 4:** Summary of studies exploring vulnerability factors associated with social anxiety in autism and interacting with Clark and Wells (1995) model

**Authors** (Year, Country)	**Sample Size** (N, male) **Recruitment Channel**	**Diagnosis** (Criteria/ Measure)	**Age** (M; SD) **IQ** (M; SD) **Ethnicity** (Caucasian N; %) **Co-occurring conditions in ASD group** (N; %)	**Social anxiety measure**	**Mechanism measure**	**Outcome**	**Quality Score**
A. ***Social Anhedonia (Social Situation, n* = *1)***							
^†^Gadow et al. (2020); USA	ASD: 268 (233) Non-ASD psychiatry referrals: 641 (447) Clinical site	Clinical diagnosis by DSM-IV AutSev: SCQ	***Age:*** ASD: 10.5 (3.3); Non-ASD: 12.2 (3.4) ***IQ* (n/% < *70*)*:*** ASD: 71 (26.4%); Non-ASD: 18 (3.1%) ***Ethnicity:*** 229 (81.5) ***Co-occurring:*** none reported	CASI-4R Social Anxiety subscale (parent)	Social motivation: CASI-4R Social Anhedonia subscale (parent)	***Covariates controlled for:*** age, gender, intellectual disability ***Social anxiety:*** ASD and non-ASD with ↑ Social Anhedonia = ↑ SA; Social anhedonia + preference to be alone > social anhedonia – preference to be alone (ASD: *d* = .53; non-ASD: *d* = .82) ***Social anhedonia:*** 32% of autistic youths with social anhedonia preferred to be alone, compared to 7% of non-autistic youths ***Interaction:*** SA contributes to odds of meeting criteria for social anhedonia even when accounting for reciprocal social interaction skills (OR = 1.46, *p* < .001) For both groups: ↑ SA = ↑ Social Anhedonia (*r* = .38, *p* < .001)	0.95
B. ***Intolerance of Uncertainty (Social Situation to Activate Assumptions, n* = *1)***							
Pickard et al. (2020); UK	ASD: 61 (42) NT: 62 (26) School	Clinical and school diagnosis *AutSev* (ASD): SRS-2 77.47 (10.65); SCQ lifetime 20.94 (6.98)	***Age****:* ASD: 13.46 (1.77); NT: 13.52 (1.57) ***IQ:*** ASD: 98.16 (13.99); NT: 100.76 (11.55) ***Ethnicity:*** none reported ***Co-occurring:*** none reported	LSAS (self)	IUS (self, caregiver)	***Covariates controlled for:*** sex ***Social anxiety:*** 52.4% of ASD vs. 54.8% NT > SA cut-off ***IU-Parent:*** ASD > NT (*r* = .49, *p* < .001) ***Interaction:*** ASD—IU-child: ↑ SA = ↑ IU (*r* = .71, *p* < .001), IU not associated with autism traits (*r* = .14, *p* > .05) NT – IU-^child^: ↑ SA = ↑ IU (*r* = .62, *p* < .001), ↑ IU = ↑ autism traits (*r* = .41, *p* < .001) ASD – IU-parent: ↑ SA = ↑ IU (*r* = .53, *p* < .001), ↑ IU = ↑ autism traits (*r* = .46, *p* < .001); IU mediated association between SA and Autistic traits NT – IU-parent: ↑ SA = ↑ IU (*r* = .40, *p* < .01), ↑ IU = ↑ autism traits (*r* = .75, *p* < .001) Stronger association between autistic traits and parent-report IU in NT vs ASD (*Z* = -2.53, *p* = .011)	0.91
C. ***Autistic Identity (Activate Assumptions, n* = *1)***							
Cooper et al., (2022a, 2022b); UK	ASD: 121 (82) Community	Clinical diagnosis; SRS-S *AutSev:* SRS-S: 18.5 (6.1)	***Age:*** 17.6 (1.1) ***IQ:*** not reported ***Ethnicity:*** 109 (90) ***Co-occurring:*** none reported	SAS-A (self)	Adapted version of Social Identification scale (Leach et al., 2008) (self)	***Covariates controlled for:*** age, gender, autism traits ***Social anxiety:*** ↑ Autism traits = ↑ SA (*r* = .57, *p* < .001) ***Autism Identity:*** ↑ Autism traits = ↑ central autism identity (*r* = .34, *p* < .001) and ↑ solidarity with other autistic people (*r* = .26, *p* < .01) ***Interaction:*** Accounting for gender, age, autism traits: ↑ Autism satisfaction = ↓ SA (ß = -0.2, p < .05) Accounting for age and gender: ↑ SA = ↑ Autism Centrality (*r* = .22, *p* < .05), not with autism solidarity (*r* = .15, *p* > .05), autism satisfaction (*r* = .19, *p* > .05), autism self-stereotyping (*r* = .15, *p* > .05), or autism homogeneity (*r* = .06, *p* > .05)	0.95
D. ***Attentional Bias / Threat Monitoring (Activating Assumption to Perceived Social Danger, n* = *8)***							
Corden et al. (2008); UK	ASD: 21 (16) Non-ASD: 21 (16) Community	Clinical diagnosis; ADOS, ADI-R, 3Di *AutSev* (ASD): ADOS: 9.8 (3.75)	***Age:*** ASD: 33.8 (13.6); Non-ASD: 32.1 (11.58) ***IQ:*** ASD: 117.9 (11.67); Non-ASD: 117.2 (8) ***Ethnicity*:** none reported ***Co-occurring:*** none reported	SPAI (self)	Ekman-Friesen test of facial affect recognition (self)	***Covariates controlled for:*** none reported; matched on age, IQ, visual-perceptual ability ***Social anxiety:*** ASD > non-ASD ***Facial Recognition:*** ASD < non-ASD: recognising fearful and sad faces ***Interaction:*** ASD: ↑ SA = ↓ fear recognition (*r* = -.51, *p* = .04), and ↓ time spent fixating on eyes (*r* = -.5, *p* = .04) Non-ASD: no significant interaction	0.82
Hollocks et al. (2013); UK^1^	ASD: 38 (38) Control A: 21 (21) Control B: 20 (20) School	Clinical diagnosis and enrolment in specialist ASD school *AutSev:* SCQ 22 (5.6)	***Age:*** ASD: 12.9 (1.4); Control A: 13.6 (1.2); Control B: 14.2 (1.8) ***IQ:*** ASD: 95.6 (13.3); Control A: 107 (9.99); Control B: 117 (8.89) ***Ethnicity:*** none reported ***Co-occurring:*** none reported	SCAS-P (caregiver) SCAS-C (self)	Attention: Dot probe emotional faces/words Emotion recognition: affect recognition & inhibition from NEPSY-II	***Covariates controlled for:*** IQ ***Social anxiety:*** ASD < Control A and Control B on parent-rated SA ***Attention:*** ASD slower than Control A and B on face and word tasks, but no difference in attention bias ***Interaction:*** Parent rated SA not associated with threat face bias (*r* = .04, *p* = .85), social word bias (*r* = .13,* p* = .53), or physical word bias (*r* = .09, *p* = .67)	0.77
Hollocks et al. (2016); UK^1^	ASD: 21 (21) ASDAnx: 34 (34) Non-ASD: 28 (28) Clinical site	Clinical diagnosis, ADI-R/ADOS, SCQ *AutSev:* SCQ 19.4 (5.7) for ASD, 24.7 (5.8) for ASDAnx	***Age:*** ASD: 13 (1.9); ASDAnx: 12.7 (1.9)***;*** Non-ASD: 13.9 (1.8) ***IQ:*** ASD: 103 (16.7); ASDAnx: 99.7 (10;9); Non-ASD: 116 (9.5) ***Ethnicity:*** none reported ***Co-occurring:*** 62% ≥ 1 anxiety; 18% ≥ 3 anxiety; 21 panic/agoraphobia, 22 GAD, 14 separation, 2 simple phobia, 4 SAD, 11 OCD	SCAS-P (caregiver) SCAS-C (self)	Attention: Dot-Probe (self) Emotion recognition: emotional face and words (self)	***Covariates controlled for:*** IQ ***Social anxiety:*** ASDAnx > ASD > non-ASD ***Attention:*** ASDAnx > ASD, non-ASD on threat-bias towards angry faces ***Interaction:*** ASD and ASDAnx groups: ↑ SA (child) = ↑ threat face bias (*r* = .40, *p* = .003) SA rated by parent and child not associated with social threat words (*r* = .05 to .08, *p* > .05), physical threat words (*r* = -.17 to -.04, *p* > .05)	0.86
*Kanat et al. (2017); Germany	ASD: 29 (29) NT: 30 (30) Clinical site	Clinical diagnosis by DSM-IV, ADOS-4 *AutSev* (ASD): AQ 37.8 (8.1)	***Age:*** ASD: 38.2 (10.6); NT: 32.1 (12.3) ***VIQ:*** no intellectual disability ***Ethnicity:*** none reported ***Co-occurring****:* 18 depression, 7 ADHD, 6 anxiety disorders	SIAS (self)	Attention: dot-probe task using face/house (self)	***Covariates controlled for:*** social anxiety symptom severity ***Intervention:*** self-administered 3 puffs of oxytocin or placebo nasal spray 45 min prior to computer task ***Interaction:*** ASD-Placebo: ↑ SA = ↓ attention to faces (*r* = -.56, *p* = .002) ASD-oxytocin: SA not associated with attention to faces (*r* = -.2, *p* = .289), significant decrease from placebo condition (*z* = -1.5, *p* = .07) NT: weak negative association between SA and attention to faces under placebo (*r* = -.03, *p* = .12), no association following oxytocin (*r* = -.56, *p* = .79)	0.82
*Kang et al. (2019); USA	ASD: 53 (38) Clinical site	Clinical diagnosis, ADOS-2 *AutSev:* none reported	***Age:*** 11.6 (2.96) ***IQ:*** 103.49 (15.4) ***Ethnicity:*** none reported ***Co-occurring:*** none reported	MASC-2-P (caregiver) MSAC-2-C (self-report)	Threat monitoring: Flanker task (error-rated negativity; ERN) (self)	***Covariates controlled for:*** baseline scores and intervention condition ***Intervention:*** 1.5 h/week ten-week RCT of a group Social Skills Intervention (treatment vs. active control). Treatment included theatre-based activities. Control included group games or projects ***Social anxiety:*** Intervention group: 39% parents and 52% young people-reported significant ↓ in SA ***Interaction:*** Baseline: ↑ SA = ↑ negative ERN (*r* = -.345, *p* = .17), ↑ negative ERN predicted ↑ improvement	0.85
*Luxford et al.(2017); UK	ASD: 35 (31) INT: 18 WLC: 17 School	Clinical diagnosis *AutSev:* SCQ INT: 18.61 (4.33) WLC: 19.06 (4.94)	***Age:*** 13.2 (1.1) ***IQ:*** INT: 105.44 (17.83); WLC: 102 (11.3) ***Ethnicity*:** none reported ***Co-occurring:*** none reported	SWQ-C (Self) SWQ-T (teacher)	Attention control: Erikson flanker task Attention to threat: Emotional stroop colour matching schematic face task (self)	***Covariates controlled for*:** baseline anxiety for pre-post analyses ***Intervention:*** ‘Exploring Feelings’ manualised CBT – 6 weekly 90 min sessions in small groups of 4–6 participants in school, weekly homework, 6-week follow-up ***Social anxiety:*** INT = WLC: self-report ↓ SA at end of intervention and follow-up INT > WLC: teacher-report ↓ SA at end of intervention and follow-up ***Attention control:*** INT < WLC on conflict score; *Attention to threat:* both groups—angry bias > happy ***Interaction:*** T1: neither self nor teacher-reported SA were associated with attention control (*r* = -.08 to -0.06, *p* > .05), attention to threat for happy bias (*r* = -0.18 to .04, *p* > .05), angry bias (*r* = -0.01 to .18, *p* > .05), fear bias (*r* = -0.09 to .21, *p* > .05)	0.79
May et al. (2015); Australia	ASD: 45 (22) NT: 45 (22) Clinical site, community	Clinical diagnosis by DSM-IV-TR, SRS *AutSev* (ASD): SRS 100.1 (29.7)	***Age:*** ASD: 10.27 (1.55); NT: 9.86 (1.55) ***VIQ:*** ASD: 99.2 (14.1); NT: 106.9 (14.3) ***Ethnicity:*** none reported ***Co-occurring:*** none reported	SCAS Social Phobia scale (Parent)	Threat bias: visual dot probe emotional faces (self)	***Covariates controlled for:*** VIQ ***Social anxiety:*** ASD group scored similarly to children with anxiety disorders (n = 333) in Nauta et al. (2004) study ***Attention/Threat bias:*** No differences between groups on threat or happy congruent/incongruent/neutral trials ***Interaction:*** No significant association between SA and facilitated or disengagement from either threat or happy faces on the dot probe task for ASD and NT group	0.86
Spain et al. (2016); UK	ASD: 50 (50) Clinical site, community (from a larger study)	Clinical diagnosis by ICD-10, ADI-R, ADOS-G, AQ *AutSev:* ADOS-G total 10.2 (4.7)	***Age****:* 26.3 (5.8) ***IQ:*** 108 (14.9) ***Ethnicity:*** none reported ***Co-occurring:*** none reported	LSAS-SR (self) BFNE (self) SPS (self) SIAS (self)	KDEF (self)	***Covariates controlled for:*** none reported ***Social anxiety:*** 26 scored above cut-off on LSAS-SR ***Interaction:*** No significant correlations between any of the SA measures and any socio-emotional tests (*r* < .24, *p* > .05). Comparing those who scored > and < SA cut-off, no differences in performances on any socio-emotional tasks	0.82
White et al. (2015); USA	ASD: 15 (8) NT: 18 (10) Clinical site, Community	Clinical diagnosis; ADOS, ADI-R *AutSev* (ASD): SRS total 87.53 (16.69)	***Age:*** ASD: 14.88 (1.55); NT: 14.33 (1.52) ***IQ:*** no ID reported by parents ***Ethnicity:*** ASD: 12 (80); NT: 17 (94.4) ***Co-occurring:*** 6 SAD	SWQ-P (caregiver) SWQ-C (self) BFNE (self)	Attention/threat-bias: gaze patterns for positive/negative face (self)	***Covariates controlled for:*** autism traits ***Social anxiety:*** ASD and non-ASD group matched ***Interaction:*** ASD: ↑ FNE (child-report) = ↑ gaze to anger (*r* = .75, *p* < .01) and ↑ gaze to disgust (*r* = .68, *p* < .01) faces, but not happy faces (*r* = .4, *p* > .05) NT: no association between FNE and gaze to anger faces (*r* = .33, *p* > .05), disgust faces (*r* = .25, *p* > .05) or happy faces (*r* = .16 *p* > .05)	0.91
E. ***Assertiveness (Fear of Negative Evaluation; n* = *3)***							
Bellini (2004); USA^2^	ASD: 41 (35) School	Clinical diagnosis *AutSev:* none reported	***Age:*** 14.22 ***FSIQ:*** 99.94 (18.81) ***Ethnicity:*** none reported ***Co-occurring:*** none reported	SAS-A (self) MASC-Social Anxiety (humiliation/performance fears) (self)	SSRS (self, caregiver)	***Covariates controlled for:*** none reported ***Social anxiety:*** 20 out of 41 > cut-off for SA; 5 out of 41 scored below low SA ***Interaction:*** • ↓ assertion = ↑ SA and distress in new (*r* = -.31, *p* < .05) and general situations (*r* = -.39, *p* < .01), but not fear of negative evaluation (*r* = -.13, *p* > .05) • ↓ assertion = ↑ performance fears (*r* = -.33, *p* < .05), but not with humiliation fears (*r* = -.25)	0.77
Bellini (2006); USA^2^	ASD: 41 (35) School	Clinical diagnosis *AutSev:* none reported	***Age:*** 14.22 ***FSIQ:*** 99.94 (18.81) ***Ethnicity:*** none reported ***Co-occurring:*** none reported	SAS-A (self)	SSRS (self, caregiver)	***Covariates controlled for:*** none reported ***Interaction:*** ↓ assertion = ↑ SA (*β* = -1.68, *p* < .01)	0.64
Chang et al. (2012)	ASD: 53 Clinical site, community, school	Clinical diagnosis; ADI-R; ADOS *AutSev:* none reported	***Age:*** 9.55 (1.73) ***IQ:*** none reported ***Ethnicity:*** none reported ***Co-occurring:*** 49 separation anxiety, 55 GAD, 54 SAD, 30 OCD	ADIS-C/P (clinician)	SSRS (parent)	***Covariates controlled for:*** none reported ***Social anxiety:*** 92.5% > cut-off ***Interaction:*** ↑ SA = ↑ social skills difficulties including assertion (*R*^*2*^ = .12) and responsibility (*R*^*2*^ = .12)	0.77
F. ***Emotion Regulation (Somatic/Cognitive Symptoms to Processing of Self as Social Object, n* = *1)***							
Swain et al. (2015); USA	ASD: 69 (49) Clinical site	Clinical diagnosis *AutSev:* SRS-2 69.67 (10.13	***Age****:* 20.5 (2) ***IQ:*** none reported ***Ethnicity:*** 41 (59.42) ***Co-occurring:*** none reported	SAS (self)	DERS (self)	***Covariates controlled for:*** none reported ***Interaction:*** ↑ caregiver rated SA = ↑ difficulty in selecting effective emotion regulation strategies (*r* = .25, *p* < .05); ↑ emotion dysregulation (*r* = .25, *p* < .05) ↑ caregiver rated FNE = ↑ non-acceptance of negative emotions (*r* = .26, *p* < .05), ↑ difficulty with goal-directed behaviours for negative emotions (*r* = .26, *p* < .05), ↑ total emotion dysregulation (*r* = .26, *p* < .05) ↑ self-rated SA = ↑ non-acceptance of negative emotions (*r* = .61, *p* < .01), ↑ inability to act in goal-directed way (*r* = .47, *p* < .01), ↑ total emotion dysregulation (*r* = .61, *p* < .01) ↑ self-rated FNE = ↑ non-acceptance of negative emotions (*r* = .54, *p* < .01), ↑difficulty with goal-directed behaviour for negative emotions (*r* = .47, *p* < .01), ↑limited access to strategies for emotion regulation (*r* = .59, *p* < .01), ↑ total emotion dysregulation (*r* = .58, *p* < .05)	0.86
G. ***Alexithymia (Emotion Regulation, n* = *5)***							
Albantakis et al. (2020); Germany	ASD: 122 (83); non-ASD: 62 (37); NT: 261 (37) Clinical site	DSM-5 criteria; ADOS-2 *AutSev:* ADOS: 7.01 (3.17) AQ: 36.25 (8.16)	***Age:*** ASD: 33.46 (10.40); non-ASD: 35.15 (12.62); NT: 26.41 (7.8) ***IQ:*** none reported ***Ethnicity****:* none reported ***Co-occurring:*** none reported	LSAS (self)	TAS-20 (self)	***Covariates controlled for:*** age, sex ***Social anxiety:*** ASD > non-ASD > NT ***Alexithymia:*** ASD > non-ASD > NT ***Interaction:*** ASD: alexithymia (step 1; *R*^*2*^ = 11.8%) no longer significantly associated with SA when accounting for autism traits (step 2; *R*^2^ = 10.3%). Autism traits (step 1; *R*^*2*^ = 21.1%) significantly associated with SA when accounting for alexithymia (step 2; *R*^2^ = 1%) Non-ASD: alexithymia (step 1; *R*^*2*^ = 30.1%) significantly associated with SA when accounting for autism traits (step 2; *R*^2^ = 3.6%). Autism traits (step 1; *R*^*2*^ = 17.4%) significantly associated with SA when accounting for alexithymia (step 2; *R*^2^ = 16.2%) NT: alexithymia (step 1; *R*^*2*^ = 17.2%) significantly associated with SA when accounting for autism traits (step 2; *R*^2^ = 6.2%). Autism traits (step 1; *R*^*2*^ = 15.5%) significantly associated with SA when accounting for alexithymia (step 2; *R*^2^ = 7.9%)	0.91
Antezana et al. (2023); USA	ASD: 19 (19) Community	ADOS-2 *AutSev:* ADOS-2 comparison: 5 (1.79)	***Age:*** 21.82 (3.83) ***FSIQ-2:*** 108.28 (16.47) ***Ethnicity:*** 16 (84.21) ***Co-occurring:*** none reported	LSAS (self)	TAS-20 (self)	***Covariates controlled for:*** age, IQ ***Social anxiety:*** 13 out of 19 > cut-off ***Alexithymia:*** 7 out of 19 > cut-off ***Interaction:*** • ↑ SA = ↓ overall facial emotion recognition accuracy, more in the low IQ (79–106) group (*r* = -.78, *p* < .01) and not in high IQ (107–14) group (*r* = -.43, *p* = .25) • SA not associated with alexithymia (*r* = .31, *p* = .197)	0.91
Pickard et al. (2020); UK	ASD: 61 (42) NT: 62 (26) School	Clinical and school diagnosis; SCQ-L and SRS-2 *AutSev* (ASD): SRS-2 77.47 (10.65); SCQ lifetime 20.94 (6.98)	***Age:*** ASD: 13.46 (1.77); NT: 13.52 (1.57) ***IQ:*** ASD: 98.16 (13.99); NT: 100.76 (11.55) ***Ethnicity:*** none reported ***Co-occurring:*** none reported	LSAS (self)	TAS-20 (self)	***Covariates controlled for:*** sex ***Social anxiety:*** 52.4% of ASD vs. 54.8% NT > SA cut-off ***Interaction:*** ASD: ↑ SA = ↑ Alexithymia (*r* = .63, *p* < .001), and ↑ Alexithymia = ↑ autism traits (*r* = .36, *p* < .01)	0.91
Ringold et al. (2022); USA	ASD: 57 (44) Developmental Coordination Disorder (DCD): 26 (15) NT: 53 (31) Clinical site, community, school	Clinical diagnosis, ADI-R, ADOS-2 *AutSev:* none reported	***Age:*** ASD: 11.89 (2.29); DCD: 11.75 (2.31); NT: 11.75 (2.13) ***IQ:*** ASD: 107.51 (16.82); DCD: 109.69 (17.13); NT: 118.28 (13.74) ***Ethnicity:*** none reported ***Co-occurring:*** none reported	SCARED-P (Caregiver)	AQC (Caregiver)	***Covariates controlled for:*** age, sex, IQ ***Social anxiety:*** ASD > DCD, NT ***Alexithymia:*** ASD > DCD, NT ***Interaction****:* ASD: ↑ sensory over-responsivity = ↑ alexithymia (*r* = .30, *p* < .01); ↑ sensory over-responsivity = ↑ SA (*r* = .47, *p* < .01) DCD: ↑Sensory over-responsivity = ↑ SA (*r* = .54, *p* < .01) No effect in TD group (*r* = .24)	0.91
Wilson et al. (2023); UK	ASD: 192 (56) Non-ASD: 69 (26) Self-identify ASD: 51 (14) Broad Aut Phenotype (BAP): 24 (9) Community Online	Clinical diagnosis; AQ AutSev (ASD): AQ-10 7.89 (1.79)	***Age:*** ASD: 40.95 (13.62); N-ASD: 40.30 (17.28); S-ASD: 41.98 (13.95); BAP: 32.82 (11.16) ***IQ:*** none reported ***Ethnicity:*** ASD: 165 (86); N-ASD: 61 (88); S-ASD: 42 (82); BAP: 17 (71) ***Co-occurring:*** 58 SAD, 36 ADHD, 13 dyslexia, 15 dyspraxia, 9 language/learning-related diagnosis	LSAS (self)	GAFS-8 (self)	***Covariates controlled for:*** none reported; no association between outcome variable sand age/gender ***Social anxiety*:** ASD > non-ASD (Cohen’s *d* = .1.01) ***Alexithymia:*** ASD > non-ASD (Cohen’s *d* = 1.28) ***Interaction:*** Alexithymia did not account for additional variance between ↑ autism traits and ↑ SA. SA and Alexithymia’s association: *r* = .18	0.95
H. ***Hyper/Hypo Sensitivity (Emotion Regulation, n* = *5)***							
^†^Black et al. (2017); Canada	ASD: 39 (30) Non-ASD: 40 (16) Clinical site, school	Clinical diagnosis; ADOS, AQ-C *AutSev* (ASD group): AQ: 97.7 (13.6)	***Age:*** ASD: 12.08 (2.63); Non-ASD: 11.03 (3.03) ***VIQ:*** ASD: 45 (13.3); Non-ASD: 51.9 (7.1) ***Ethnicity:*** none reported ***Co-occurring:**** none reported*	SCAS-P (caregiver)	SSP (caregiver)	***Covariates controlled for:*** gender, VIQ, PIQ ***Social anxiety:*** No difference between ASD and non-ASD groups ***Interaction:*** Hypersensitivity not associated with SA in ASD group (*r* = .26, *p* > .05) nor TD group (*r* = .07, *p* > .05)	0.86
Ludlow et al. (2023); UK	ASD + Selective Mutism (SM): 38 (21) SM: 37 (11)	Clinical diagnosis (n = 32) or referred for ASD diagnosis (n = 6) *AutSev* (ASD): AASQ 23.76 (9.26)	***Age:*** SM + ASD: 9 (4.39); SM: 10.51 (4.25) ***IQ:*** none reported ***Ethnicity:*** none reported ***Co-occurring:*** 38 selective mutism	RCADS-P Social Anxiety subscale (caregiver)	SP2 (caregiver)	***Covariates controlled for:*** GAD symptom severity ***Social anxiety:*** SM + ASD > SM ***Sensory sensitivity:*** SM + ASD > SM on sensory seeking, sensitivity, avoidance, and low registration ***Interaction:*** Group differences on SA partially mediated by levels of sensory avoidance (*β* = .47, *p* < .001), even when controlling for GAD	0.77
MacLennan et al. (2020); UK	ASD: 41 (28) Clinical site, community, online	Clinical diagnosis of ASD by DSM-IV or DSM-5; AQ *AutSev:* AQ 35.5 (5.8)	***Age:*** 8.44 (2.86) ***IQ:*** 109.03 (16.92) ***Ethnicity:*** none reported ***Co-occurring:*** none reported	SCAS-P (caregiver) PAS (caregiver)	SPSI (caregiver)	***Covariates controlled for:*** autism symptom severity; age and IQ did not affect significance of associations ***Social anxiety:*** 35 (62.9%) showed elevated symptoms ***Interaction:*** When controlling for autism severity, ↑ SA = ↓ hyporeactivity (*Spearman’s rho* = -.397, *p* < .05); SA not associated with hyperreactivity (*Spearman’s rho* = .08, *p* > .05)	0.86
Pickard et al. (2020); UK	ASD: 61 (42) NT: 62 (26) School	Clinical and school diagnosis; SCQ-L and SRS-2 *AutSev* (ASD): SRS-2 77.47 (10.65); SCQ lifetime 20.94 (6.98)	***Age:*** ASD: 13.46 (1.77); NT: 13.52 (1.57) ***IQ:*** ASD: 98.16 (13.99); NT: 100.76 (11.55) ***Ethnicity:*** none reported ***Co-occurring:*** none reported	LSAS (self)	AASP (self)	***Covariates controlled for:*** sex ***Social anxiety:*** 52.4% of ASD vs. 54.8% NT > SA cut-off ***Hyposensitivity:*** NT > SAD (*d* = .41, *p* < .05) ***Interaction:*** ASD-Hypersensitivity: ↑ SA = ↑ hypersensitivity (*r* = .62, *p* < .001), ↑ SA = ↑ autistic traits (*r* = .33, *p* < .05), hypersensitivity mediated association between SA and autistic traits NT-Hypersensitivity: ↑ SA = ↑ hypersensitivity (*r* = .67, *p* < .001), ↑ SA = ↑ autistic traits (*r* = .36, *p* < .01) ASD-Hyposensitivity: ↑ SA = ↑ hyposensitivity (*r* = .29, *p* < .05) NT-Hyposensitivity: no association between hyposensitivity and SA (*r* = .09) nor autistic traits (*r* = -.09)	0.91
Ringold et al. (2022); USA	ASD: 57 (44) Developmental Coordination Disorder (DCD): 26 (15) NT: 53 (31) Clinical site, community, school	Clinical diagnosis, ADI-R, ADOS-2 *AutSev:* none reported	***Age:*** ASD: 11.89 (2.29); DCD: 11.75 (2.31); NT: 11.75 (2.13) ***IQ:*** ASD: 107.51 (16.82); DCD: 109.69 (17.13); NT: 118.28 (13.74) ***Ethnicity:*** none reported ***Co-occurring:*** none reported	SCARED-P (Caregiver)	SSP-2 (Caregiver) SensOR (Caregiver)	***Covariates controlled for:*** age, sex, IQ ***Social anxiety:*** ASD > DCD, NT ***Sensory differences:*** ASD > DCD, NT on sensory seeking (partial η^2^ = .32), avoidance (partial η^2^ = .46), sensitivity (partial η^2^ = .51), oversensitivity on tactile (partial η^2^ = .39) and auditory (partial η^2^ = .36) ***Interaction:*** ASD: ↑Sensory over-responsivity = ↑ SA (*r* = .47, *p* < .01), ↑ Alexithymia (*r* = .30, *p* < .01) DCD: ↑Sensory over-responsivity = ↑ SA (*r* = .54, *p* < .01), not Alexithymia (*r* = .18) NT: No effect between sensory over-responsivity and SA (*r* = .24), nor Alexithymia (*r* = -.17)	0.91
I. ***Camouflaging (Safety Behaviours and Processing of Self as Social Object, n* = *4)***							
Hull et al. (2019); UK^3^	ASD: 354 (108) Non-ASD: 478 (192) Clinical site, Community	Clinical diagnosis *AutSev:* BAPQ	***Age:*** ASD: 41.93 (13.55); Non-ASD: 30.24 (13.72) ***IQ:*** none reported ***Ethnicity****:* none reported ***Co-occurring:*** none reported	LSAS (self)	CAT-Q (self)	***Covariates controlled for:*** none reported ***Interaction:*** In ASD group: ↑ SA = ↑ camouflaging (*r* = .44, *p* < .001), ↑ compensation (*r* = .30, *p* < .001), ↑ masking (*r* = .19, *p* < . 01), ↑ assimilation (*r* = .60, *p* < .001) In non-ASD group: ↑ SA = ↑ camouflaging (*r* = .60, *p* < .001), ↑ compensation (*r* = .46, *p* < .001), ↑ masking (*r* = .35, *p* < . 001), ↑ assimilation (*r* = .69, *p* < .001)	0.86
Hull et al. (2021); UK^3^	ASD: 305 (104) Clinical site	Clinical diagnosis *AutSev:* BAPQ 4.32	***Age:*** 41.9 ***IQ:*** none reported ***Ethnicity:*** none reported ***Co-occurring:*** 25% have 1 dx, 14% with 2 dxs, 9% ≥ 3 dxs; 173 GAD, 166 depression, 7 SAD	LSAS (self)	CAT-Q (self)	***Covariates controlled for:*** age, autistic traits ***Interaction:*** Social camouflaging accounted for additional variance in SA symptom severity in a linear fashion (*β* = .21, *p* < .001), relationship did not differ by gender identity	0.91
Lei et al. (2023); UK	ASD: 61 (17) NT: 54 (11) Clinical site, School/University; Online	Clinical diagnosis *AutSev* (ASD): AQ-28 77.93 (10.23)	***Age:*** ASD: 16.34 (1.69); NT: 16.02 (1.56) ***IQ:*** none reported ***Ethnicity:*** ASD: 50 (81.97); NT: 37 (68.52) ***Co-occurring:*** 9 ADHD, 22 GAD, 19 SAD, 21 OCD, 1 Panic, 2 PTSD, 17 Depression, 6 Eating disorder	SPIN (self)	CAT-Q (self)	***Covariates controlled for:*** GAD and depression symptom severity, groups matched on SA ***Social anxiety:*** ASD and Non-ASD matched ***Social camouflaging:*** ASD and Non-ASD groups did not differ on compensation and assimilation; ASD < non-ASD on masking ***Interaction:*** ASD: ↑ SA = ↑ camouflaging total (*r* = .51, *p* < .001), ↑ compensation (*r* = .47, *p* < .001), ↑ assimilation (*r* = .61, *p* < .001), but not with masking (*r* = .25, *p* = .051)	0.91
Wilson et al. (2023); UK	ASD: 192 (56) Non-ASD: 69 (26) Self-identify ASD: 51 (14) Broad Aut Phenotype (BAP): 24 (9) Community Online	Clinical diagnosis; AQ *AutSev* (ASD): AQ-10 7.89 (1.79)	***Age:*** ASD: 40.95 (13.62); N-ASD: 40.30 (17.28); S-ASD: 41.98 (13.95); BAP: 32.82 (11.16) ***IQ:*** none reported ***Ethnicity:*** ASD: 165 (86); N-ASD: 61 (88); S-ASD: 42 (82)***;*** BAP: 17 (71) ***Co-occurring:*** 58 SAD, 36 ADHD, 13 dyslexia, 15 dyspraxia, 9 language/learning-related diagnosis	LSAS (self)	CAT-Q (self)	***Covariates controlled for:*** none reported; no association between outcome variable sand age/gender ***Social anxiety*:** ASD > non-ASD (Cohen’s *d* = 1.01) ***Camouflaging:*** ASD > non-ASD (Cohen’s *d* = 1.10) ***Interaction:*** Adding camouflaging did not explain additional variance between ↑ autism traits and ↑ SA. Camouflaging and SA’s association: *r* = .35	0.95

### Data Synthesis

**Fig. 2:**
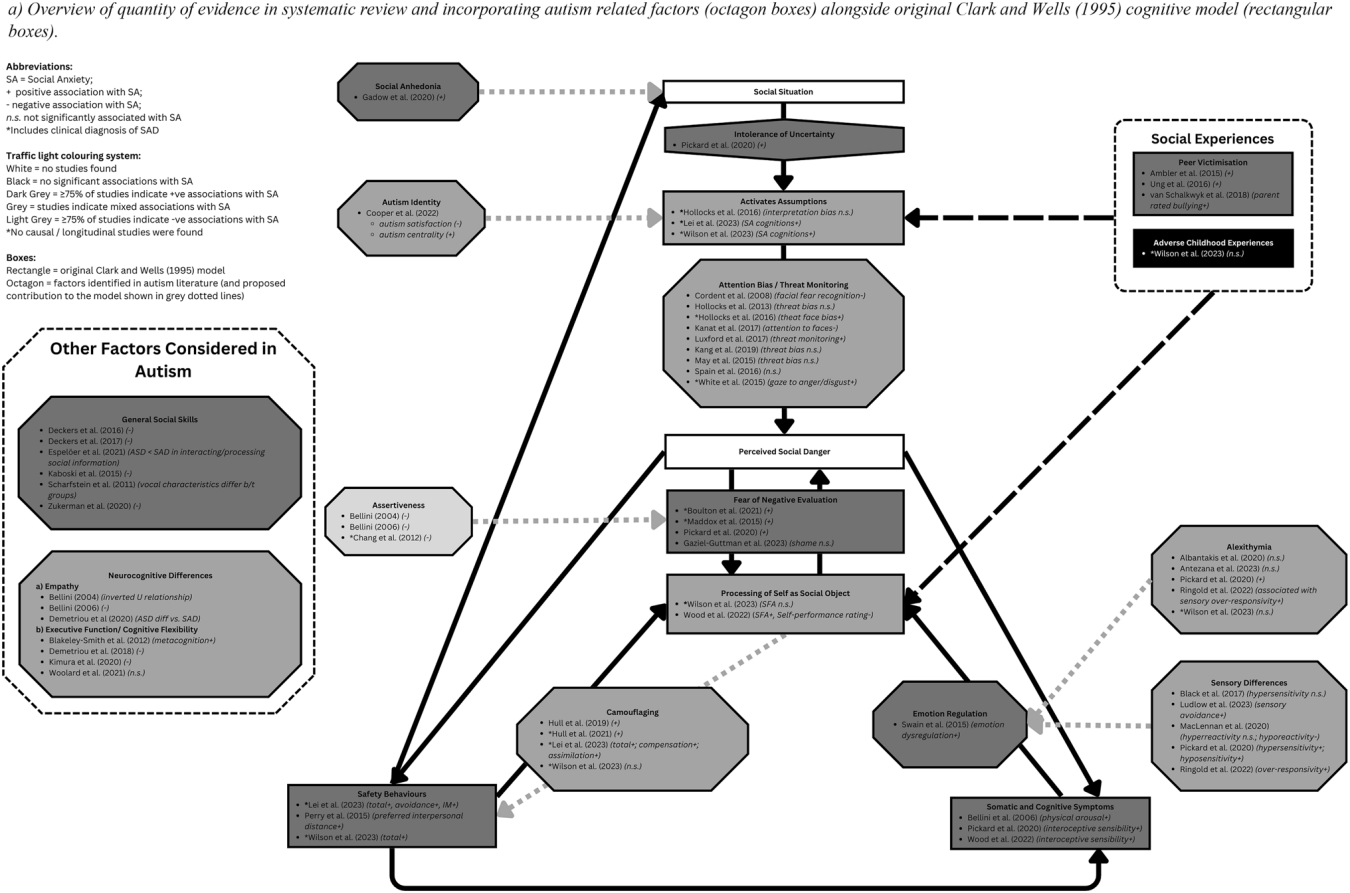

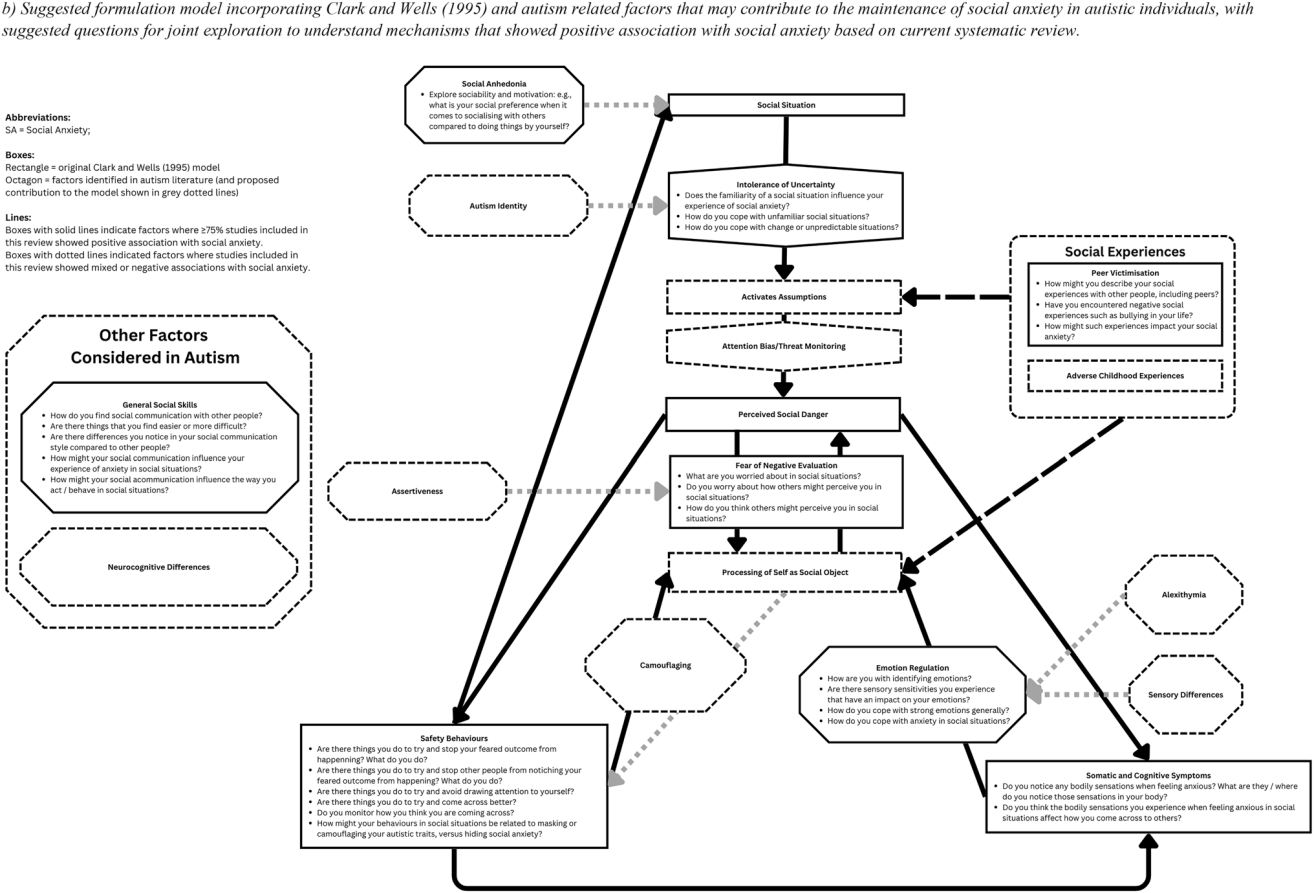
**a** Overview of quantity of evidence in systematic review and incorporating autism related factors (octagon boxes) alongside original Clark and Wells (1995) cognitive model (rectangular boxes). **b** Suggested formulation model incorporating Clark and Wells (1995) and autism related factors that may contribute to the maintenance of social anxiety in autistic individuals, with suggested questions for joint exploration to understand mechanisms that showed positive association with social anxiety based on current systematic review. All questions serve as topic guide suggestions only, and we encourage clinicians to adapt the language and framing to suit the individual needs of autistic clients they are working with

Given the heterogeneity in the range of SA and mechanism measures used across studies, we chose to synthesise data without using a meta-analysis by following guidelines outlined by Campbell et al. (2020). The first author read and re-read all included paper to familiarise themselves with data from each study relevant to the two research questions, before extracting data that included a measure of effect size indicating the interaction between SA and the mechanism investigated (e.g. Pearson’s correlation coefficient) and any group comparison (e.g. Cohen’s *d*). Data was grouped into themes according to (1) studies that investigated key mechanisms maintaining SA as outlined in the original Clark and Wells (1995) model; (2) additional factors that may interact with mechanisms outlined in the cognitive model of SA that have been explored in autistic individuals; (3) additional neurocognitive differences associated with autism that have shown association with SA. All authors agreed on the refinement of the final subthemes to categorise data before narrative synthesis. The direction of associations was summarised in Fig. 2 that show overall state of positive and negative associations between mechanisms measured and SA across studies within each subtheme. All authors approved the final synthesis.

## Results

### Overview of Included Studies

In total, 45 studies were included in the review (see Fig. 1). Most studies were conducted in the USA (*n* = 15, 33%) and UK (*n *= 14, 31%), followed by Australia (*n* = 6, 13%), Israel (*n* = 3, 7%), Germany (*n* = 3, 6%), the Netherlands (*n* = 2, 4%), Canada (*n* = 1, 2%) and Japan (*n* = 1, 2%). All studies were published between 2004 and 2023. For studies that made use of the same recruitment sample within the same research group (10 studies were identified that came from 5 independent samples), we included the study that had the largest number of participants to avoid duplication in participant demographic information.

### Quality Appraisal

Report of full quality appraisal for all included articles is available upon request from authors. No studies were excluded based on quality appraisal, though several methodological issues should be considered. On average across the studies, 66% of the participants were males, and studies mostly included participants without co-occurring intellectual disability (IQ: M = 103, SD = 14). Only 11 studies reported ethnicity data, and on average 77% of participants across these studies identified as White/Caucasian. Only 14 studies reported co-occurring conditions of autistic participants (see Table 2 for breakdown of stated diagnoses). Most participants were children and young people, with 26 studies (23 independent samples) involved a largely paediatric sample with mean age below 18 years old, and only 7 studies (6 independent samples) reported average age of participants that exceeded 30 years old. No studies employed a longitudinal design, and direction of causation between SA symptoms and mechanisms measured cannot be inferred from cross-sectional correlational measures.

For SA symptoms, the majority of studies used specific self- and/or parent/caregiver reports such as the Social Anxiety Scale (SAS; La Greca et al., 2015), Social Phobia Inventory (SPIN; Johnson et al., 2006) and Liebowitz Social Anxiety Scale (LSAS; Liebowitz, 1987). SAS-Adolescent measure has been shown to have reasonable psychometric validation in autistic adolescents though lacks measurement invariance (Schiltz et al., 2019). Amongst autistic adults without intellectual disabilities, SPIN, LSAS, Social Interaction Anxiety Scale, Social Phobia Scale, and Brief Fear of Negative Evaluation Scale have shown good internal consistency and reasonable psychometric properties (Boulton & Guastella, 2021). Only four studies used the Anxiety Disorders Interview Schedule for DSM IV (ADIS-IV, Silverman et al., 1996), which is a standardised clinician-administered diagnostic tool for SA (Chang et al., 2012; Demetriou et al., 2018, 2020; Maddox & White, 2015), though agreement between the parent/carer and young person versions of the ADIS-IV showed poor agreement amongst autistic youths (Storch et al., 2012). 34 studies used self-report measures to examine SA and the mechanism under investigation, and it is unclear the extent to which associations reported may be inflated due to reporter bias. Only 8 studies used a second informant (clinician, caregiver, or teacher) to report on SA compared to 19 studies for mechanism measurement.

### Participant Characteristics

A total of 2739 participants were recorded across the 45 included studies from 40 independent samples, with most participants recruited from clinical sites (*n* = 30 studies) and the community setting (*n* = 21 studies). Only two studies explicitly stated the inclusion of autistic participants with co-occurring intellectual disability (Black et al., 2017; Gadow & Garman, 2020). Detailed breakdown of participant characteristics for studies included to address each research question is shown in Table 2.

### Review Question 1: Research Evidence Underpinning Clark and Wells’ (1995) Cognitive Model of Social Anxiety

A total of 13 studies including 901 autistic participants (see Table 2) assessed SA symptom severity alongside a mechanism outlined in the Clark and Wells’ (1995) Cognitive Model of SA. Figure 2 shows the high-level overview summary of direction of associations reported across studies examining each maintenance factor, and results are summarised in Table 3. Greater fear of negative evaluation from others (Boulton & Guastella, 2021; Maddox & White, 2015; Pickard et al., 2020), use of safety behaviours (Lei et al., 2023; Perry et al., 2015; Wilson & Gullon-Scott, 2023), peer victimisation (Ambler et al., 2015; Ung et al., 2016; van Schalkwyk et al., 2018), and use of internal information such as somatic symptoms (Bellini, 2006; Pickard et al., 2020; H. Wood et al., 2022) were positively associated with greater SA. In contrast, negative cognitions and perceived social danger (Hollocks et al., 2016; Lei et al., 2023; Wilson & Gullon-Scott, 2023) and processing of self as a social object (Wilson & Gullon-Scott, 2023; H. Wood et al., 2022) showed mixed associations with SA. One study that examined adverse childhood experiences did not find significant association with SA (Wilson & Gullon-Scott, 2023).

#### Negative Social Cognitions

Amongst both autistic adolescents (mean age: 16 years) (Lei et al., 2023) and autistic adults (mean age: 41 years) (Wilson & Gullon-Scott, 2023), greater endorsement of SA related cognitions (e.g. “I will be frozen with fear”, “People will make fun of me”) was associated with greater SA. When matched at the group level on SA symptom severity, autistic and non-autistic adolescents showed no between group differences in SA related cognitions (Lei et al., 2023). Autistic adults showed greater SA symptom severity compared to non-autistic adults and greater endorsement of SA related cognitions (Wilson & Gullon-Scott, 2023). Using the Ambiguous Situation Interview (Barrett et al., 1996) to examine interpretation bias amongst autistic children and adolescents (mean age: 13 years), Hollocks et al. (2016) found although autistic children with co-occurring anxiety difficulties showed greater negative interpretations for social situations than non-autistic peers, interpretation bias in both social and physical situations were not associated with SA rated by parents or autistic young people themselves. Initial findings form the three studies (Hollocks et al., 2016; Lei et al., 2023; Wilson & Gullon-Scott, 2023) suggest that in both autistic adolescents and adults, greater SA related cognitions are associated with greater SA, with no evidence supporting the role of negative interpretation bias in maintaining SA. However, it is unclear to what extent items on Social Cognitions Questionnaire, especially those that relate to other’s people’s reaction in social situations, may be an accurate representation of an autistic individual’s lived experience versus an overestimation of the likelihood of negative appearance and a negative reaction from others. The items in the Ambiguous Situation Task, especially in social scenarios, may require a certain level of social communication skills to understand the context from the limited information provided, and it is unclear whether autistic young people showed similar social comprehension to their non-autistic peers, and how differences in social comprehension (rather than social anxiety) may have influenced the more negative interpretations generated by autistic young people (Hollocks et al., 2016), and were associated with general levels of anxiety in social situations, rather than specifically social anxiety per se.

#### Perceived Social Danger (including Fear of Negative Evaluation from others)

Three studies (Boulton & Guastella, 2021; Maddox & White, 2015; Pickard et al., 2020) used the self-rated Brief Fear of Negative Evaluation (BFNE) questionnaire to explore the association between social anxiety and one’s worries of how others might perceive the self in social situations (e.g. “I am afraid that others will not approve of me.”). Autistic adolescents (mean age: 13 years) who showed similar levels of SA symptoms as non-autistic peers also showed no between group differences in FNE, and FNE was positively associated with SA in both groups (Pickard et al., 2020). FNE in both autistic adults (mean age: 24 years) and adults with a clinical diagnosis of SAD showed positive associations with SA, including greater avoidance, social interaction anxiety, and being scrutinised by others (Boulton & Guastella, 2021). Compared to adults with a diagnosis of SAD, autistic adults with greater levels of SA showed greater social interaction anxiety, including “making eye contact”, “mixing comfortably with peers” and “making friends of my own age” (Maddox & White, 2015). All three studies (Boulton & Guastella, 2021; Maddox & White, 2015; Pickard et al., 2020) using the BFNE suggested that greater fear of negative evaluation is associated with greater SA in autistic children and young adults, and comparable to non-autistic peers, with two studies including young adults with social anxiety diagnosis. No studies employed qualitative methods to explore how autistic individuals interpreted items on the BFNE or scale, as some items may be interpreted from a stance of one’s own autism acceptance and identity, and experiences of stigma associated with one’s autistic identity. For example, the item “I am afraid that people will find fault with me” may tap into autistic individual’s experiences of having social communication differences pointed out to them by non-autistic individuals, rather than purely from a SA perspective (Davies et al., 2023).

A fourth study used the Guilt and Shame Proneness Scale (T. R. Cohen et al., 2011) to explore how engagement in negative social situations influences one’s emotional self-evaluation and behavioural withdrawal (Gaziel-Guttman et al., 2023). Example items include: “You lie to people, but they never find out about it. What is the likelihood that you would feel terrible about the lies you told?” and “Your home is very messy and unexpected guests knock on your door and invite themselves in. What is the likelihood that you would avoid the guests until they leave?”. Compared to non-autistic peers, autistic young adults (mean age: 24 years) showed greater social anxiety (including fear and avoidance), and greater social avoidance was associated with reduced negative self-evaluation (Gaziel-Guttman et al., 2023). Greater shame was only associated with more SA related fear in non-autistic group, but greater social withdrawal in both groups (Gaziel-Guttman et al., 2023). Given that the study did not assess nor control for autistic young adults’ understanding of the expected social outcome in each shame-arousing situation, it may be that items on the Guilt and Shame scale did not elicit internal shame in the same way for autistic and non-autistic young people. The interaction between FNE and self-evaluation in relation to experiences of SA may also be less situation specific, and more related to the individual’s general perceived sense of social acceptability considering their autistic identity, which none of the studies explicitly assessed.

#### Processing of Self as Social Object (Self Focussed Attention)

Using the Focus of Attention Questionnaire (Woody, 1996), self-focussed attention is measured by items such as “I was focusing on what I would say or do next” and “I was focusing on past social failures”, and other-focussed attention is assessed by items such as “I was focusing on other person’s appearance or dress” and “I was focusing on how the other person might be feeling about himself/herself”. Greater self-focussed attention was associated with SA in autistic adolescents (mean age: 18 years) (H. Wood et al., 2022) but not in autistic adults (mean age: 41 years) ( Wilson & Gullon-Scott, 2023). Given that the adult sample had high levels of co-occurring mental health conditions compared to absence of reporting co-occurring conditions in the adolescent study, it is unclear how co-occurring mental health difficulties other than SAD may have influenced the association between self-focussed attention and social anxiety.

#### Somatic and cognitive symptoms

Regarding using internal information to generate perception of self in the social situation, physical arousal reported by autistic adolescents (mean age: 14 years) (Bellini, 2006) and interceptive sensibility (i.e. awareness of internal bodily states rather than perception accuracy) in autistic adolescents (mean age: 13 to 18 years) (Pickard et al., 2020; H. Wood et al., 2022) were both positively associated with SA, and the latter is comparable to that found in non-autistic same-aged peers (Pickard et al., 2020). Greater interoceptive sensibility specifically related to bodily temperature, muscle tension, and heart rate were associated with greater SA, and this fully mediated the association between poorer self-rated social performance in light of greater SA (H. Wood et al., 2022). Three studies (Bellini, 2006; Pickard et al., 2020; H. Wood et al., 2022) in autistic adolescents support the hypothesis that greater awareness of internal bodily information is associated with greater SA, though no studies were found in autistic children or adults.

#### Safety Behaviours

Using the Social Behaviours Questionnaire (SBQ; Clark, 2003) which asks individuals to rate how often they do each behaviour when feeling anxious in or before a social situation, with avoidance behaviours including “avoiding talking about yourself; position yourself so as not to be noticed” and impression management behaviours including “check that you are coming across well; try to fit in and ‘act normal”, autistic adolescents (mean age: 16 years) (Lei et al., 2023) and adults (mean age: 41 years) (Wilson & Gullon-Scott, 2023) showed increased SA when engaging in greater self-report levels of safety behaviours, though the strength of the association was weaker for autistic adults compared to non-autistic adults (Wilson & Gullon-Scott, 2023). For autistic adolescents, self-report levels of avoidance and impression management behaviours were comparable to non-autistic peers who were matched on SA at the group level (Lei et al., 2023). Greater use of safety behaviours, including both avoidance and impression management, are associated with greater SA in autistic adolescents and adults (Lei et al., 2023; Perry et al., 2015; Wilson & Gullon-Scott, 2023). However, it is unclear to what extent some of the items on the SBQ are specific to anxiety related behaviours, as items such as ‘try to fit in and ‘act normal’’ may also be associated with autistic individuals’ masking behaviours to try and hide autism related social communication differences in a social situation (Hull et al., 2017).

One study used the Interpersonal Distance: Stop-Distance Paradigm (Greenberg et al., 1980) which asks participants to report when they feel “slightly or considerable discomfort” when the Experimenter is approaching them from afar, and found that autistic young adults (mean age: 25 years) showed similar mean preferred distance compared to non-autistic peers matched on SA, though greater SA was associated with greater preferred interpersonal distance in autistic young adults only (Perry et al., 2015). However, tolerance for interpersonal distance may also be affected by unexamined factors such as sensory hyper/hyposensitivity, which may moderate the association between preferred distance and SA.

#### Social Experiences (Peer victimisation and adverse childhood experiences)

Amongst autistic children (mean age 12 years old), peer victimisation (including relational, overt, and cyberbullying) over the past 12 months rated by both child self-report and caregiver report were associated with greater social avoidance, but not FNE from others (Ung et al., 2016). Amongst autistic adolescents (mean age: 16 years), only parent-rated bullying was associated with greater SA rated by both parents and adolescent self-report; and adolescent-rated bullying was not associated with SA reported by neither parents nor adolescents themselves (van Schalkwyk et al., 2018), despite adolescents reporting greater bullying compared to their caregivers. It may be that those who show greater SA are subjected to observable forms of bullying that parents are more aware of, and greater cyberbullying that parents may be less aware of. Using teacher-reported school based social behaviours, autistic adolescents (mean age: 15 years) who experienced greater SA showed greater social avoidance in class and were more likely to be ignored/rejected by peers, though social problems was not associated with social anxiety amongst non-autistic peers (Ambler et al., 2015). Overall, all three studies (Ambler et al., 2015; Ung et al., 2016; van Schalkwyk et al., 2018) suggest that experiences of peer victimisation may be associated with SA in autistic children and adolescents, especially when such experiences are reported by caregiver or teacher reports. In contrast, adverse childhood experiences (e.g. “as a child, an adult humiliated, embarrassed or scared me”) reported by autistic adults (mean age: 41 years) was not associated with SA in adulthood (Wilson & Gullon-Scott, 2023). The developing adolescent brain may be particularly influenced by quality of peer interactions (Blakemore & Robbins, 2012), and the influence of both positive and negative peer interactions on SA may be particularly pertinent during this developmental phase.

### Review Question 2: Additional mechanisms associated with SA in autistic individuals beyond the Clark and Wells’ (1995) model

A total of 37 studies (from 32 independent samples) including 2248 autistic participants (see Table 2) assessed SA symptom severity alongside mechanisms (beyond autism symptom severity) often discussed to be associated with anxiety in autism, and not directly captured by the Clark and Wells’ (1995) model. A proposed model to highlight additional factors that have been explored in autistic individuals in addition to the Clark and Wells’ (1995) Cognitive Model of SA is shown in Fig. 2. Such identified factors include social anhedonia, intolerance of uncertainty, autistic identity, attention bias and threat monitoring, assertiveness, emotion regulation, hyper-hyposensitivity, alexithymia, and camouflaging (Table 4). We also found several studies examined neurocognitive factors associated with SA amongst autistic individuals, including empathy and executive function differences. Finally, we discuss the association between general social skills (beyond autism specific social communication differences) and social anxiety amongst autistic individuals (Table 5).

**Table 5:** Summary of studies exploring general social skills and neurocognitive differences associated with social anxiety in autism

**Authors** (Year, Country)	**Sample Size** (N, male) **Recruitment Channel**	**Diagnosis** (Criteria/ Measure)	**Age** (M; SD) **IQ** (M; SD) **Ethnicity** (Caucasian N; %) **Co-occurring conditions in ASD group** (N; %)	**Social anxiety measure**	**Mechanism measure**	**Outcome**	**Quality score**
A. ***General Social Skills (n* = *7)***							
*Deckers et al. (2016); Netherlands^4^	ASD: 52 (47) Waitlist: 26 (23) Soc Skills: 26 (24) Clinical site	Clinical diagnosis by DSM-IV-TR *AutSev:* none reported	***Age:*** ASD: 10.1 (1.27); Waitlist: 10 (1.1); Soc Skills: 10.2 (1.43) ***IQ:*** not reported ***Ethnicity:*** none reported ***Co-occurring:*** 30 has co-occurring diagnosis, including 21 with ADHD	SCARED (caregiver)	SSO (caregiver, teacher)	**Intervention:** 12 weekly 1 h child sessions and three 1 h parent sessions. Each Social Skills Group has 4 autistic children, delivered by psychologist + co-therapist ***Covariates controlled for:*** none reported ***Social skills:*** Soc Skills > WLC on parent/teacher ratings of social skills improvement ***Interaction:*** ↑SA = ↓social skills (*β* = -2.56, *p* = .009). SA did not moderate change in social skills rated by teachers or parents	0.83
Deckers et al. (2017); Netherlands^4^	ASD: 73 (62) ADHD: 76 (54) Non-clinical: 106 (62) Clinical site, community, school	Clinical diagnosis by DSM-IV-TR *AutSev* (ASD): none reported	***Age:*** ASD: 11.22 (2.42); ADHD: 11.79 (2.48); Non-clinical: 11.61 (2.63) ***IQ:*** > 70 ***Ethnicity:*** predominantly Caucasian ***Co-occurring:*** 1 adjustment disorder; 17 ADHD; 9 anxiety disorders; 2 eating disorders; 4 learning disorders; 1 mood disorders; 23 relational problems; 2 other disorder or diagnosis deferred	SCARED (caregiver)	SSO (caregiver, teacher) WSIS (caregiver, teacher)	***Covariates controlled for:*** gender ***Social anxiety:*** ASD > ADHD, Non-clinical ***Social skills:*** ASD < ADHD, non-clinical (parent/teacher ratings) ***Interaction:*** ASD: ↑SA = ↓social skills rated by parents (*r* = -.47, *p* < .001) Clinical control: ↑SA = ↓social skills rated by parents (*r* = -.46, *p* < .001) Non-clinical control: no significant effect (*r* = -.47)	0.91
Espelöer et al. (2021); Germany	ASD: 23 (17) SAD: 68 (28) NT: 25 (10) Clinical site, community	Clinical diagnosis by ICD-10 *AutSev*: none reported	***Age:*** ASD: 44 (10.55); SAD: 37 (10); NT: 38.8 (10.41) ***IQ:*** ASD: 118.65 (15.58); SAD: > 80***;*** NT: 110.28 (14.11) ***Ethnicity:*** none reported ***Co-occurring:*** none reported	SASKO—SA subscale (speaking and being in focus of attention; rejected by others) (self)	Social Skills: SASKO—Social Competence (interaction deficits, deficits in processing of social information) (self)	***Covariates controlled for:*** gender ***Social anxiety:*** No group differences between ASD and SAD, both groups > NT on SA ***Social skills:*** ASD > SAD in difficulties in interaction and processing of social information (*p* < .001)	0.82
*Kaboski et al. (2015); USA	ASD: 8 (8) Non-ASD: 8 (8) Community	Clinical diagnosis by DSM-5, ADOS-2, SCQ *AutSev* (ASD): SCQ-lifetime 17.9 (4.7)	***Age:*** ASD: 14.05 (1.73); Non-ASD: 13.83 (1.45) ***IQ:*** ASD: 106 (18.56); Non-ASD: 112 (10.77) ***Ethnicity:*** none reported ***Co-occurring:*** none reported	SASC-R (self) SAS-A (self)	SSIS (parent)	**Intervention:** Two consecutive weeklong camps (3 h/day for 5 consecutive days) including career skills and robotics instructions, programming, and pair work ***Covariates controlled for:*** none reported, groups matched on age, gender, grade in school, IQ and language skills ***Social anxiety:*** ASD > non-ASD (*d* = 1.1), especially in SAD-General subscale (*d* = 1.57) ASD: significant ↓ in SA post-intervention (*d* = .74) ***Social skills:*** ASD < non-ASD on social skills (*d* = 2.7) at baseline ASD: no significant changes in social skills post-intervention (*d* = .17) ***Interaction:*** At baseline: ↓ social skills = ↑SA (*r* = -.55, *p* < .05), ↑SA in new situations (*r* = -.54, *p* < .05), ↑SA in general situations (*r* = -.59, *p* < .05), though not with fear of negative evaluation (*r* = -.40, *p* > .05)	0.79
Scharfstein et al. (2011); USA	ASD: 30 (26) SAD: 30 (23) NT: 30 (22) Clinical site	Clinical diagnosis;, ADI-R *AutSev:* none reported	***Age:*** ASD: 10.57 (1.6); SAD: 10 (1.8)*;* NT: 10.6 (2) ***IQ:*** ASD: 114 (14.08) ***Ethnicity:*** ASD: 27 (90); SAD: 18 (60)***;*** NT: 11 (36.7) ***Co-occurring:*** none reported	SPAI-C (self) SAM (self)	BAT (observer)	***Covariates controlled for:*** race (though results did not differ with/without covariate) ***Social anxiety:*** SAD > ASD, NT (partial η^2^ = .18) on self-reports and blind observer ratings (partial η^2^ = .18) ***Social behaviours:*** • SAD < ASD, NT on overall social skills (partial η^2^ = .12) • SAD < NT on pragmatic social behaviours (partial η^2^ = .12) • SAD < ASD, NT on appropriate speech and prosodic social behaviours (partial η^2^ = .17) • SAD and ASD < NT on vocal intensity (partial η^2^ = .43) • SAD < ASD and NT on vocal intensity variability (partial η^2^ = .18) • SAD > ASD on vocal pitch (partial η^2^ = .08) and vocal pitch variability (partial η^2^ = .09) ***Interaction:*** Vocal characteristics significantly contributed towards predicting group membership when accounting for observer-rated social skill differences (effect size = .98)	0.92
Zukerman et al. (2020); Israel	ASD: 53 (49) University	Clinical diagnosis of ASD; AQ *AutSev:* AQ 43.79 (4.5)	***Age:*** 23.53 (2.81) ***IQ:*** WAIS comprehension 87.97 (9.18) ***Ethnicity:*** none reported ***Co-occurring:*** none reported	LSAS (self)	Social skills: Comprehension/Social adaptive behaviour gap (C-SOC) (self)	***Covariates controlled for:*** age ***Interaction:*** ↑ SA = ↓ social adaptive behaviours (*r* = -.42, *p* < .01), and ↑ gap between comprehension and social adaptive behaviours (*r* = .40, *p* < .01) ↑SA avoidance = ↑ C-SOC gap (*β* = .49, *p* < .001)	0.77
B. ***Neurocognitive Differences (n* = *7)***							
a) ***Empathy (n* = *3)***							
Bellini (2004); USA^2^	ASD: 41 (35) School	Clinical diagnosis *AutSev:* none reported	***Age:*** 14.22 ***FSIQ:*** 99.94 (18.81) ***Ethnicity:*** none reported ***Co-occurring:*** none reported	SAS-A (self) MASC-Social Anxiety (humiliation/performance fears) (self)	SSRS (self, caregiver)	***Covariates controlled for:*** none reported ***Social anxiety:*** 20 out of 41 > cut-off for SA; 5 out of 41 scored below low SA ***Interaction:*** Empathy shows curvilinear (inverted U) relationship with Fear of Negative Evaluation (η^2^ = .40), and distress in new (η^2^ = .27), general (η^2^ = .33) situations, and performance fears (η^2^ = 0.19)	0.77
Bellini (2006); USA^2^	ASD: 41 (35) School	Clinical diagnosis *AutSev:* none reported	*Age:* 14.22 *FSIQ:* 99.94 (18.81) *Ethnicity:* none reported *Co-occurring:* none reported	SAS-A (self)	SSRS (self, caregiver)	***Covariates controlled for:*** none reported ***Interaction:*** ↓ empathy = ↑ SA (*β* = -13.35, *p* < .01)	0.64
Demetriou et al. (2020)^5^	ASD: 62 (41) SAD: 83 (55) Early Psychosis: 48 (22) NT: 43 (22) Clinical site, community	Clinical diagnosis; ADOS-2 and ADI-R *AutSev:* ASD Group: SRS-2 Total = 72.64 (13.23) ASD + SAD Group: SRS-2 Total = 71.96 (13.12)	***Age:*** ASD: 22.63 (5.55); SAD: 22.34 (6.15); EP: 23.08 (5.76); NT: 23.21 (5.84) ***IQ:*** > 70 ***Ethnicity:*** none reported ***Co-occurring:*** none reported	ADIS-IV/V (clinician)	EQ (self)	***Covariates controlled for:*** none reported, no group differences in age/gender ***Empathy:*** ASD and SAD groups distinguished by overall levels of empathy	0.91
b) ***Executive Function (including cognitive flexibility) (n* = *4)***							
Blakeley-Smith et al. (2012); USA	ASD: 63 (56) Clinical site	Clinical diagnosis; ADOS, ADI-R, SCQ *AutSev:* none reported	***Age:*** 10.83 (1.72) ***VIQ:*** 107 (16.3) ***Ethnicity:*** 54 (85.7) ***Co-occurring:*** none reported	SCARED-P (caregiver); SCARED-C (self)	BRIEF (caregiver)	***Covariates controlled for:*** none reported ***Social anxiety:*** Child and parent ratings showed non-significant, though moderate levels of agreement (*r* = .59) ***Interaction:*** Metacognitive ability significantly correlated with parent–child agreement on the SA domain (*r* = .33)	0.86
Demetriou et al. (2018); Australia^5^	ASD: 60 (38) SAD: 76 (41) Early Psychosis: 58 (37) NT: 59 (31) Clinical site, community	Clinical Diagnosis; ADOS *AutSev:* none reported	***Age:*** ASD: 24.11 (7.27); SAD: 22.11 (5.64); EP: 21.79 (4.05); NT: 24.88 (5.3) ***IQ:*** ASD: 107.56 (8.66); SAD: 111.28 (6.56); EP: 100.71 (9.37); NT: 106.99 (8.3) ***Ethnicity:*** none reported ***Co-occurring:*** none reported	ADIS (clinician)	Cognitive flexibility: CANTAB-IED, TMT-B, COWAT, BRIEF (self)	***Covariates controlled for:*** IQ, education ***Cognitive Flexibility:*** ASD > EP, SAD and NT on EF impairments, including psychomotor speed, mental flexibility, sustained attention, and fluency tasks	0.91
Kimura et al. (2020); Japan	ASD: 33 (24) Non-ASD: 35 (21) Clinical site	Clinical diagnosis by DISCO *AutSev (ASD):* SRS 112.18 (31.56)	***Age:*** ASD: 27.63 (6.18); Non-ASD: 28.03 (5.88) ***VIQ:*** ASD: 105.94 (11.04); Non-ASD: 106.46 (11.43) ***Ethnicity:*** none reported ***Co-occurring:*** none reported	LSAS (self)	Cognitive flexibility: letter fluency (self)	***Covariates controlled for:*** none reported, matched on FSIQ and VIA ***Social anxiety:*** ASD > non-ASD on total, fear and avoidance SA scores ***Cognitive flexibility:*** ASD < non-ASD on total number of correct responses ***Interaction:*** ASD: ↑ correct responses on letter fluency = ↓SA (*Spearman’s rho* = -.38, *p* = .029) Non-ASD: ↑ correct responses on letter fluency = ↓SA (avoidance) (*Spearman’s rho* = -.491, *p* = .003), ↓SA (fear/anxiety) (*Spearman’s rho* = -.542, *p* = .001)	0.82
Woolard et al. (2021); Australia	ASD: 62 (40) Employed: 57 Not employed: 5 Clinical site	Clinical diagnosis by DSM-5, ADOS-2 *AutSev:* ADOS-2 9.71 (2.75)	***Age****:* 23.27 (6.79) ***IQ:*** 107.17 (10.07) ***Ethnicity:*** 59 born in Australia ***Co-occurring:*** none reported	LSAS (self)	Cognitive flexibility: TMT-B; BRIEF-A (self)	***Covariates controlled for:*** IQ, ADOS-2, employment status, age ***Interaction:*** SA not associated with cognitive flexibility (*r* = .22, *p* > .05)	0.86

*AASP* Adolescent/Adult Sensory Profile, *ABAS-II* Adaptive Behaviour Assessment System, *ADHD* Attention Deficit Hyperactivity Disorder, *ADI* Autism Diagnostic Interview, *ADIS-C/P* Anxiety Diagnostic Interview Schedule – Child/Parent, *ADOS* Autism Diagnostic Observation Schedule, *AQ* Autism Quotient, *AQC* Alexithymia Questionnaire for Children, *ASD* Autism Spectrum Disorder, *BAPQ* Broader Autism Phenotype Questionnaire, *BAT* Behavioural Assessment Task, *BFNE* Brief Fear of Negative Evaluation Questionnaire, *BRIEF* Behaviour Rating Inventory of Executive Function, *CANTAB IE/D* CANTAB Intra/Extra Dimensional Set Shifting task, *CASI-4R* Child and Adolescent Symptom Inventory – 4R, *CAT-Q* Camouflaging of Autistic Traits Questionnaire, *COWAT* Controlled Oral Word Association Test, *C-SOC* diff between WAIS-III comprehension and ABAS-II social adaptive behaviour, *DERS* Difficulties in Emotion Regulation Scale, *3Di* Developmental, Dimensional and Diagnostic Interview, *Dx(s)* diagnosis, *EQ* Empathy Quotient, *FSIQ* Full Scale IQ, *GAD* Generalised Anxiety Disorder, *GAFS-8* General Alexithymia Factor Score, *INT* Intervention, *IRI* Interpersonal Reactivity Index, *IUS* Intolerance of Uncertainty Scale, *KDEF* Karolinska Directed Emotional Faces, *LSAS* Liebowitz Social Anxiety Scale, *MASC(-2-P/C)* Multidimensional Anxiety Scale for Parent/Children, *NT* Neurotypical, *OCD* Obsessive Compulsive Disorder, *PAS* Preschool Anxiety Scale, *PTSD* Post Traumatic Stress Disorder, *QAQ-YA* Quality of Socialisation Questionnaire – Young Adult, *RCADS-P* Revised Children’s Anxiety and Depression Scale-Parent, *SAD* Social Anxiety Disorder, *SAS-(A)* Social Anxiety Scale (for adolescents), *SAM* Self-Assessment Manikin, *SAS-A* Social Anxiety Scale for Adolescents, *SASC-R* Social Anxiety Scale for Children-Revised, *SASKO* Social Anxiety – Social Competence Deficit Scale, *SCARED-P* Screen for Child Anxiety Related Emotional Disorders-Parent, *SCAS-P/C* Spence Children’s Anxiety Scale – Parent/Child, *SCQ* Social Communication Questionnaire, *SIAS* Social Interaction Anxiety Scale, *SP2* Sensory Profile-2, *SPAI* Social Phobia and Anxiety Inventory, *SPAI-C* Social Phobia and Anxiety Inventory – Child, *SPIN* Social Phobia Inventory, *SPSI* Sensory Processing Scale Inventory, *SPS* Social Phobia Scale, *SRS-(S)* Social Responsiveness Scale-(Short), *SSIS* Social Skills Improvement System, *SSO* Social Skills Observation, *SSP* Short Sensory Profile, *SSRS* Social Skills Rating Scale, *SWQ-C/Y* Social Worries Questionnaire – Child/Teacher, *TAS-20* Toronto Alexithymia Scale, *TMT-B* Trail Making Test-B, *WLC* Waitlist control

* Intervention study

^†^Including individuals with intellectual disability

^1,2,3,4,5^ Studies that accessed participants from the same recruitment sample are labelled with the same number

#### Social Anhedonia

Social anhedonia describes the reduced ability to derive pleasure from social interactions that can increase social withdrawal and a preference to be alone. It is considered to be a transdiagnostic symptom and has been associated with more severe autistic symptoms, as well as greater levels of SA (Gadow & Garman, 2020). One study explored the associations between social anhedonia, SA, and autistic traits in a group of autistic and non-autistic children (mean age 10–12 years), and found that greater social anhedonia in both groups was associated with greater SA, and 32% of autistic youths with social anhedonia preferred to be alone compared to 7% of non-autistic children (Gadow & Garman, 2020). Being one of the only studies to include and control for co-occurring intellectual disability, Gadow and Garman (2020) suggest that autistic children may have greater preference to be alone compared to non-autistic children in the context of social anhedonia.

#### Intolerance of Uncertainty (IU)

The negative of uncertainty (i.e. IU) contributes to the development and maintenance of anxiety in autistic (Boulter et al., 2014) and non-autistic (Boelen et al., 2010) children and adolescents. In the context of SA, IU may be associated with greater negative appraisal of ambiguous social cues. One study used the self- and caregiver report versions of the Intolerance of Uncertainty Scale (IUS) (Boulter et al., 2014; e.g. “People should always think about what will happen next. This will stop bad things from happening.”) to explore IU in autistic adolescents (mean age: 13 years) and non-autistic adolescents with comparable SA (Pickard et al., 2020). Both parent and adolescent-rated IU were significantly associated with greater SA in both groups, though parent-rated and adolescent-rated IU mediated the association between SA and autistic traits for autistic and non-autistic adolescents, respectively, highlighting the importance of gathering multi-informant report when working with adolescents (Pickard et al., 2020). IU has previously been hypothesised as a key factor that mediates the relationship between restricted and repetitive patterns of behaviours and interests (core autistic trait), and anxiety symptoms in autistic individuals (Boulter et al., 2014), and may be an important maintenance factor for SA amongst autistic adolescents (Pickard et al., 2020).

#### Autistic identity

Autistic identity refers to how an autistic individual identifies with their diagnosis, and is a multi-faceted construct that carries both a positive valence (such as accepting and viewing one’s autistic identity to be important and positive, eliciting connecting with and perceived similarity to other autistic individuals), and a negative valence (such as rejecting one’s identity, feeling helpless as a result of being autistic, and dissociation from other autistic individuals) (Davies et al., 2023). Cooper et al. (2022b) explored the association between SA and different aspects of autistic identity in autistic adolescents (mean age: 17.6 years), including measures of solidarity (extent of connection to other autistic people, e.g. “I feel a social bond with autistic people”), satisfaction (positive perception of one’s autistic identity, e.g. “I am glad to be autistic”), centrality (importance of one’s autistic identity, e.g. “Being autistic is an important part of how I see myself”), individual self-stereotyping (how similar one feels to other autistic people, e.g. “I have a lot in common with the average autistic person”), and in-group homogeneity (whether individual perceives autistic people to be similar to one another, e.g. “autistic people have a lot in common with each other”). Greater SA was associated with greater autism centrality when controlling for age and gender and with poorer autism satisfaction when controlling for autistic traits (Cooper et al., 2022a, 2022b). Autistic adolescents who perceive their autistic identity to be important but less positively may experience greater SA (Cooper et al., 2022a, 2022b). There may be a reciprocal relationship between an autistic adolescent’s awareness of their own social communication differences and FNE from others. It may be that having a negative social autistic identity may activate one’s own negative attitudes and cognitions about how one may come across in social situations and increase likelihood for adopting a negative interpretation bias in social situations.

#### Attention Bias/Threat Monitoring

Common measures for threat monitoring and attention bias detection included visual dot probe tasks such as using different emotional faces or words (Hollocks et al., 2013, 2016; Kanat et al., 2017; May et al., 2015; Ozsivadjian et al., 2021), facial affect and emotion recognition tasks (Corden et al., 2008; Hollocks et al., 2013; Spain et al., 2016), the attention control tasks such as gaze patterns for positive / negative faces (White et al., 2015), and the Erikson flanker task to inhibit responses to irrelevant stimuli under different conditions (e.g. happy versus angry faces) (Kang et al., 2019; Luxford et al., 2017). Responses in autistic children (mean age across studies: 10–15 years) showed mixed findings, such that longer time spent gazing at anger/disgust correlated with greater FNE (White et al., 2015), and a bias towards threatening faces was associated with greater SA (Hollocks et al., 2016). Other studies found contradictory evidence where parent and teacher rated SA were not associated with autistic children’s bias towards threatening faces or words (Hollocks et al., 2013, 2016; Luxford et al., 2017). Three studies that involved autistic adults (mean age 36–38 years) found that mixed findings where greater SA was associated with reduced fear recognition and time spent fixating on eyes (Corden et al., 2008), reduced attention paid to faces in general (Kanat et al., 2017), and no significant associations with performance on facial emotion recognition task (Spain et al., 2016).

It is important to contextualise the mixed findings given that one meta-analysis found autistic individuals to show poorer facial emotion recognition than other clinical populations, and task-specific variable such as emotion complexity and holistic processing of facial stimuli can affect autistic individuals’ task performance (Yeung, 2022). However, the meta-analysis found that although such differences exist when comparing group means with non-autistic individuals, there is a huge degree of individual differences across studies in autistic individuals’ task performance, which was unaccounted for by heterogeneity in mean age, sex, IQ, symptom severity and psychiatric comorbidity. This further suggests that methodological differences may play a larger role in task performance, and the processing of fear versus happy face contrasts may largely depend on characteristics of the participants and the task stimuli used, and individual differences reflect both differences in basic perception of faces as well as facial emotion processing (Yeung, 2022). Relating back to the cognitive model, we hypothesise that threat monitoring and facial emotion processing differences may influence one’s perception of danger in social situations, though no studies have explicitly tested whether facial emotion processing moderates the association between activating negative social cognitions and perceived social danger in autistic individuals, and little can be concluded from the mixed findings reported to date that have only looked at the association between SA and facial emotion processing.

#### Assertiveness

Three studies looked at the association between assertiveness measured by Social Skills Rating Scale (SSRS) and SA in two independent samples of autistic children (mean age: 10–14 years), and found that greater SA reported by autistic young people and parent/clinician was associated with poorer assertiveness reported by autistic young people and parents (Bellini, 2004, 2006; Chang et al., 2012). Given that items on the assertion scale reflect approach behaviours in social situations to either initiate contact with peers or seek help form adults, the negative associations with SA measure may reflect direct contrast to social avoidance behaviours. Approach behaviours through assertion may counterbalance avoidance behaviours driven by SA.

#### Emotion and Sensory Processing

##### Emotion Regulation

One study examined the association between emotion regulation and SA symptoms in autistic young adults (mean age: 21 years) (Swain et al., 2015) by using the Difficulties in Emotion Regulation (DERS) self-report questionnaire (Gratz & Roemer, 2004). Both parent and self-reported SA were associated with greater emotion dysregulation rated by young adults, with parent-reported SA associated with more difficulties in selecting effective emotion regulation strategies, and young people-reported SA associated with greater non-acceptance of negative emotions and inability to act in a goal-oriented way (Swain et al., 2015). Both parent and self-reported FNE were also associated with total emotion dysregulation, non-acceptance of negative emotions and difficulty with goal-directed behaviours for negative emotions, and young people rated SA was associated with more limited access to strategies for emotion regulation (Swain et al., 2015).

##### Alexithymia

Five studies explored the association between alexithymia and SA, with two in autistic children and adolescents (mean age: 12–13 years) (Pickard et al., 2020; Ringold et al., 2022) and three in adults (mean age: 22–41 years) (Albantakis et al., 2020; Antezana et al., 2023; Wilson & Gullon-Scott, 2023). All studies used variations of the Toronto Alexithymia Scale (TAS-2) that look at difficulties with identifying and describing feelings (Bagby et al., 1994). Studies found that self-reported SA was associated with greater alexithymia in autistic adolescents (Pickard et al., 2020), but not in young adults (Antezana et al., 2023) or adults when accounting for autistic traits (Albantakis et al., 2020; Wilson & Gullon-Scott, 2023). Alexithymia was also associated greater sensory over-responsivity in autistic adolescents, which in turn was associated with greater caregiver-reported SA (Ringold et al., 2022). Inconsistent results in alexithymia highlight potential construct overlap between alexithymia and autistic traits which includes preference for analytical thinking and difficulties in social emotion processing.

##### Sensory Differences

Five studies explored the association between SA and sensory processing differences in autistic children (mean age across studies: 8–13 years), drawing primarily on caregiver reports using the Sensory Profile (Black et al., 2017; Ludlow et al., 2023; Ringold et al., 2022) or Sensory Processing Scale Inventory (MacLennan et al., 2020), with only one study looked at autistic children’s self-report using the Adolescent version of Sensory Profile (Pickard et al., 2020). Greater SA reported by caregivers were shown to be associated with reduced sensory hyporeactivity (MacLennan et al., 2020), though mixed findings with sensory hyperreactivity with one suggesting greater over-responsivity (Ringold et al., 2022) and two studies suggesting no associations (Black et al., 2017; MacLennan et al., 2020). Only one study looked at the association between autistic adolescents’ self-reported SA and sensory processing differences, and found that greater SA was associated with both greater hyper- and hyposensitivity, and hypersensitivity also mediated the association between SA and autistic traits (Pickard et al., 2020). Although no studies explicitly assessed the association between alexithymia and sensory processing differences and emotion regulation, we hypothesise that both perceptual differences in sensory experiences and the ability to describe and identify emotions may contribute towards one’s awareness of emotions and ability to engage in emotion regulation strategies appropriately. Awareness, identification, and selection of effective emotion regulation strategies may influence how individuals process somatic and cognitive symptoms of SA to form an internal image of how one comes across in social situations.

#### Camouflaging

Four studies explored the association between social camouflaging and SA, with three focusing on autistic adults (mean age across studies: 41–42 years) (Hull et al., 2019, 2021; Wilson & Gullon-Scott, 2023), and one on autistic adolescents (mean age: 16 years) (Lei et al., 2023). Camouflaging describes a range of behaviours that autistic individuals may endorse when hiding their autistic traits and trying to fit in to the neurotypical environment (Hull et al., 2017). All studies used the self-report Camouflaging Autistic Traits Questionnaire (CAT-Q; Hull et al., 2019), which encompasses three subscales: (1) Assimilation – behaviours used to fit in with others and not stand out from the crowd; (2) Compensation – behaviours used to compensate for autism-related difficulties in social situation; (3) Masking – behaviours used to hide autistic characteristics or present a non-autistic personality. Greater self-reported SA was found to be associated with greater camouflaging behaviours overall, as well as greater compensation and assimilation in both autistic adolescents (Lei et al., 2023) and adults (Hull et al., 2019), though masking was only significantly associated with SA in autistic adults (Hull et al., 2019). Mixed findings emerged when accounting for autistic traits, with one study showing camouflaging accounted for additional variance in SA reported by autistic adults (Hull et al., 2021), and another suggesting a null effect (Wilson & Gullon-Scott, 2023). Self-report measures of camouflaging may capture “camouflaging intent” or conscious efforts to employ such strategies in social situations, rather than “camouflaging efficacy” or success in meeting the intended aim of hiding autistic traits (Cook et al., 2021).

Some behaviours on the CAT-Q bear resemblance to impression management behaviours that form part of the safety behaviours maintaining social anxiety over time, and it is unclear how autistic individuals who experience higher levels of SA may be interpreting and reporting such items, whether the self-monitoring involved and putting an ‘act to fit in’ is specific to autistic traits or resulting from social anxiety and FNE from others (Cook et al., 2021; Fombonne, 2020). Only one study explored simultaneous associations between autistic traits, SA, safety behaviours and camouflaging in autistic and non-autistic adolescents matched on SA, and found that masking behaviours and impression management behaviours were both significantly associated with SA rather than autistic traits (Lei et al., 2023). We therefore hypothesise that camouflaging behaviours may influence the association between safety behaviours and processing of self as a social object within the cognitive model of SA.

#### Other factors considered in autism in relation to social anxiety

##### General Social Skills (beyond core autism specific social communication differences)

Six studies looked at the association between SA and social skills differences not measured by autistic trait or characterisation questionnaires, but rather on social skills demonstrated in a social interaction context such as via the Social Skills Observation (SSO; Barry et al., 2003), Social Skills Improvement System (SSIS; Gresham & Elliott, 2008) and role-play based tasks such as the Behavioural Assessment Task (Beidel et al., 1999). Given that the original Clark and Wells (1995) cognitive model of SA assumed the absence of underlying social interaction skills impairments, we wanted to explore how autistic individuals may display differences in general social skills and adaptive social behaviours during social interactions, rather than social communication differences assessed as part of core autistic traits. When looking at the association between SA and social skills reported by caregivers or teachers amongst autistic children and adolescents (mean age across studies: 10–14 years), greater SA was associated with poorer social skills during interactions (Deckers et al., 2016, 2017; Kaboski et al., 2015; Scharfstein et al., 2011), and vocal characteristics during role-play based task helped observers differentiate autistic children from children with SAD and non-autistic children without SA (Scharfstein et al., 2011). Two studies with autistic adults found that SA in autistic young adults (mean age: 24 years) was associated with poorer social adaptive behaviours (e.g. “invites others home for fun activity”; Gray & Carter, 2013) and an increase in gap between social comprehension and social adaptive behaviours (Zukerman et al., 2020). Autistic adults (mean age: 44 years) showed greater difficulties in social interaction and social information processing compared to those with SAD (Espelöer et al., 2021).

Given that the associations between SA and autistic traits have been summarised in another systematic review (Spain et al., 2018), the current study focussed on social interaction based skills reported via non-autistic trait based questionnaires, and found that the majority of studies suggested autistic adolescents and adults demonstrated poorer social interaction skills when experiencing greater social anxiety. However, it should be noted that studies only controlled for demographic variables such as age, race, and gender of participants or no covariates at all, and not for social communication difficulties related to autistic traits that may confound the results. There may be social skills difficulties (not just autistic traits) that may underlie SA reported by autistic individuals, and the assumption from the original Clark and Wells (1995) model that there are no social skills deficits underlying one’s social performance does not hold for autistic individuals.

##### Neurocognitive differences

Three studies drawing on two independent samples looked at the association between SA and empathy in autistic children (mean age: 14 years) (Bellini, 2004, 2006), and autistic young adults (mean age: 23 years) (Demetriou et al., 2020). Using the Empathy subscale from the Social Skills Rating Scale (e.g. “understanding how friends feel” and “feel sorry for others”), Bellini (2004, 2006) found that autistic children with greater social anxiety showed poorer empathy, and empathy also showed a curvilinear relationship (inverted U) with FNE and distress in general and new social situations, as well as performance fears. Using the Empathy Quotient which looks at how one may be in tune with how other people are thinking and feeling (e.g. “I really enjoy caring for other people”), autistic young adults reported lower levels cognitive empathy, emotional reactivity and overall levels of empathy that differentiated them from non-autistic individuals with SAD (Demetriou et al., 2020).

Four studies also looked at the association between SA and parent-reported executive function in autistic children (mean age: 11 years) (Blakeley-Smith et al., 2012), and performance on tasks assessing cognitive flexibility in autistic adults (mean age across studies: 23–28 years) (Demetriou et al., 2018; Kimura et al., 2020; Woolard et al., 2021). Using the caregiver report form of the Behaviour Rating Inventory of Executive Function (BRIEF), greater agreement on parent and child reported SA was associated with metacognitive abilities, which includes executive function skills such as initiation, working memory, planning, organisation, and monitoring (Blakeley-Smith et al., 2012). For autistic adults, using a combination of self-report version of the BRIEF and tasks such as letter fluency and set-shifting, mixed results emerge where one study found cognitive flexibility is associated with lower self-reported SA (Kimura et al., 2020), another showing a null effect when controlling for autistic traits (Woolard et al., 2021). Compared to non-autistic adults with social anxiety disorder, autistic adults show greater impairments in mental flexibility (Demetriou et al., 2018). No studies that assessed empathy controlled for the potential confounding effects of autistic traits when examining associations with SA, especially when breaking empathy into its cognitive and affective components which may differentially be associated with autistic traits (Song et al., 2019). Although there is some evidence to suggest that cognitive flexibility is associated with internalising and externalising behaviours in autistic children and adolescents (Lei et al., 2022), none have looked at the specificity of this association in the context of SA per se, and one systematic review that looked at the association between cognitive flexibility and co-occurring psychological difficulties in autistic adults also found no significant associations with SA (St. John et al., 2022).

## Discussion

CT-SAD is recommended by NICE (National Institute for Health & Care Excellence, 2013) as first-line treatment for social anxiety in non-autistic adults, with evidence also emerging in the treatment of adolescents (Leigh & Clark, 2018). Building upon Spain and colleagues' (2017) review that outlined general adaptations to facilitate autistic individuals to access CBT for social anxiety, and their subsequent (2018) review that looked at core autistic traits in relation to social anxiety, this systematic review set out to review the state of evidence in autistic individuals across the lifespan for the association between social anxiety and maintenance mechanisms specifically identified in the Clark and Wells (1995) model. The current review aimed to identify research gaps where evidence is missing, as well as enabling clinicians working to support autistic individuals with SA to make informed and evidence-based decisions during assessment and formulation to inform treatment planning.

In response to review question one, there is some evidence in support of maintenance factors of social anxiety identified in the Clark and Wells (1995) model in autistic individuals, namely the impact of peer victimisation on the experience of social anxiety, greater fear of negative evaluation from others, the use of somatic symptoms and safety behaviours were all associated with greater self-reported social anxiety. However, the activation of social anxiety related cognitions and processing of self as a social object by drawing on internal information presents more mixed findings. In response to review question two, additional mechanisms associated with autism show that social anhedonia, intolerance of uncertainty, poor emotion regulation, and poor general social skills (beyond autism specific social communication difficulties) were all positively associated with greater self-reported social anxiety. Based on our findings, we outline a proposed model incorporating additional vulnerability factors identified within the autism literature to highlight mechanisms that clinicians may wish to assess and formulate with autistic clients when drawing on the Clark and Wells (1995) model of social anxiety (Fig. 2b). We also provide some suggested questions for clinicians to explore together with their clients, to better understand the interaction between original maintenance factors proposed by Clark and Wells (1995), and additional autism related vulnerabilities, for factors where ≥ 75% of the evidence evaluated within the current review suggested positive association with social anxiety (Fig. 2b). We hope clinicians can directly draw on the evidence presented in this systematic review in their assessment and formulation process to explore with autistic clients their experience of social anxiety in the context of autism.

Overall, we found that the quantity and quality of evidence supporting constructs identified in the Clark and Wells (1995) model is limited, and all proposed interactions between additional autism related vulnerability factors and the original maintenance factors are conjectural, as no studies employed a longitudinal or prospective design that simultaneously explored all the associated factors in a way that would allow direction of causation to be inferred. Few studies reported or controlled for co-occurring mental health conditions such as generalised anxiety and depression, and some of the cognitive (e.g. negative self-appraisal in social situations) and behavioural (e.g. safety behaviours including social avoidance and impression management) mechanisms may not be uniquely associated with SA, but also be influenced by co-occurring levels of depression (e.g. persistent low mood and anhedonia, Russell et al., 2020) and generalised anxiety (e.g. greater intolerance of uncertainty, Dugas, 2004).

Given that most studies employed self-report questionnaires to measure both SA and mechanisms, it is important to highlight that few explicitly assessed the psychometric properties of such questionnaires in autistic individuals, when the majority have been developed and normed in neurotypical individuals without considering potential construct overlap between autistics traits and mechanism measured. In addition, it is important to note that there may be construct overlap at the individual item level between autism related processes (such as camouflaging) and social anxiety maintenance factors (such as safety behaviours). For example, questionnaires such as the CAT-Q have been originally developed based on autistic women’s qualitative experiences of masking and camouflaging autistic traits, but the acts of monitoring one’s behaviour and consciously trying to come across well may also show construct overlap with impression management safety behaviours in SA context outside of autism, and therefore autistic individuals using such questionnaires may conflate between different constructs and result in inflated correlations across measures. For example, one study found that when simultaneously accounting for autistic traits and SA in a sample of autistic and non-autistic adolescents, camouflaging behaviours such as masking (i.e. hiding one’s autistic traits) showed greater construct overlap with impression management safety behaviours and both were significantly associated with SA rather than autistic traits (Lei et al., 2023).

Finally, we draw our attention to the one study in the current review that specifically set out to explore the Clark and Wells model alongside autism related factors when applied to autistic and non-autistic adults (Wilson & Gullon-Scott, 2023), which found that the Clark and Wells variables accounted for half of the variance associated with social fears, thus suggesting additional autism related factors need to be considered when developing person-centred formulation with autistic individuals who experience SA. Extending beyond a former systematic review that evaluated the association between core autistic traits and SA (Spain et al., 2018), we found that greater general social skills difficulties assessed from social interactions by using non autism-diagnostic/autistic trait questionnaires were associated with greater SA, and no studies explored how the interaction between general social skills and quality of social experiences may influence how autistic individuals form assumptions about their social acceptance by others, and cognitive assumptions they hold about how they may come across in social situations. Some inferences about autistic people’s perception of self may be drawn from autistic identity literature, where greater environmental support and acceptance of neurodivergence (i.e. such as peer inclusion, family support, and positive social experiences) can increase one’s ability to form a positive sense of self and one’s autistic identity, and peer support such as connecting with similar others and peer mentoring schemes may also encourage connectedness with the wider autistic community (Davies et al., 2023).

## Generalisability of Findings

Across studies, limitations on several aspects of participant demographic variables limits the generalisability of current findings to autistic individuals across the spectrum. Firstly, most studies have included autistic participants without co-occurring intellectual disability, and it remains unclear how SA may present in autistic individuals with co-occurring intellectual disability, and how co-occurring intellectual disability may affect certain social cognitive and behavioural mechanisms that are hypothesised to maintain SA from the Clark and Wells (1995) model. Most studies included involved autistic children, adolescents, and young people, and no studies involved autistic individuals with a mean sample age of greater than 42 years old. Of the few studies that reported ethnicity information, most participants identified as White/Caucasian. Few studies reported co-occurring conditions. The characterisation of autistic participants beyond autistic symptoms, age, and intellectual disability therefore remained relatively poor across studies, and future studies can benefit from recruiting autistic individuals from a more diverse and representative background, as well as consistently recording, and reporting demographic information for characterisation.

Finally, most participants self-identified as male, and the influence of gender identity on experiences of social anxiety in autistic individuals need further exploration. For example, non-autistic women are more likely to report greater clinical severity of SA and report fear of a greater range of social situations, and are less likely to seek treatment compared to men (Asher et al., 2017). It is helpful to contextualise such findings alongside gender differences in camouflaging behaviours when comparing autistic females and males, where autistic females often report greater levels of camouflaging behaviours to ‘fit in’ with neurotypical peers by hiding their autistic traits that may contribute towards late autism diagnosis (Cook et al., 2021). Camouflaging behaviours are also shown to have construct overlap with impression management behaviours, potentially both maintained by increased self-focussed attention and monitoring to ensure that one is coming across well in social situations, and are related to greater levels of social anxiety rather than autistic traits (Lei et al., 2023). Furthermore, no studies looked at the experience of SA in non-binary autistic individuals or other forms of gender identity, and there is evidence that non-autistic adults from transgender/non-binary gender identity subgroups report elevated levels of SA compared to cis-gender male and females (Mahon et al., 2023). Given that autistic individuals are more likely to be transgender compared to non-autistic adults (van der Miesen et al., 2018) and experience greater distress when navigating a largely neurotypical and cis-gender world with greater barriers to accessing healthcare (Cooper et al., 2022a, 2022b), future studies can explore how SA may manifest in gender-diverse autistic individuals, and how gender-diversity as well as biological sex may interact with cognitive, behavioural, and autism-related vulnerability factors that maintain SA over time.

## Limitations and Considerations

A wider limitation of the current systematic review is that we only included quantitative studies, thus excluding qualitative accounts of autistic individuals’ experiences of SA that might offer context to some of the mechanism associations identified. Given the focus was specifically on SA and related mechanisms in autistic individuals, we excluded studies that used an anxiety total score or internalising symptom score if specific associations with SA were not reported and authors did not respond to our request for unpublished data. The lack of participants with co-occurring intellectual disability may also reflect the fact that we did not include intellectual disability in our search term, and autistic individuals may have formed a subgroup in studies that looked at presentation of SA in those with intellectual disabilities.

## Research Implications

Future studies should consider employing longitudinal designs or experimental manipulation tasks that allow inferences to be made about how mechanisms outlined in the Clark and Wells (1995) cognitive model and additional factors identified in autism literature can serve as a predisposing, perpetuating, or even a protective factor when interacting with SA over time. Given potential construct overlap across measures of autistic traits, SA, and mechanisms outlined in the current study, future studies need to pay closer attention to psychometric validation when using self-report questionnaires in autistic individuals, ideally gather multiple informant reports where possible, and contextualise quantitative findings with qualitative reports on how such questions may be interpreted by autistic individuals to ensure that the items have good construct validity and specificity before interpreting results. The use of both questionnaires based social skills assessment and performance-based tasks to explore social interaction skills in real-time when accounting for level of autistic traits would be crucial to help understand the relationship between social skills and SA in autistic individuals, beyond core autistic traits. To highlight additional gaps identified in literature and directions for future research, we contextualise current findings by referring to the list of testable hypotheses outlined by Clark (2005) generated from the Clark and Wells (1995) Cognitive Model of Social Anxiety (see Box A).

**Box A:** .

**Hypotheses for individuals with greater SA (**Clark, 2005**)**	**Current Evidence (Section)**	**Suggested Future Research Directions**
H1. Interpretation of external social events in an excessively negative fashion	RQ1 S1 RQ1 S5 RQ2 S2 RQ2 S4	• Explore how SA related social cognitions and interpretation bias may be associated with autistic individuals’ lived experiences and social comprehension skills • Explore whether intolerance of uncertainty may uniquely contribute towards activating negative interpretation bias and biased assumptions about one’s own performance in social situations when accounting for its associations with symptoms of generalised anxiety and non-social specific worries • Explore real-time interpretation of external social cues using experimental paradigms in addition to questionnaire-based outcome measures
H2. Greater self-focussed attention when anxious in social situations	RQ1 S3	• Examine the relationship between autistic identity and fear of negative evaluation amongst autistic individuals and explore whether this association may be partially mediated by one’s negative social attitudes, cognitions, and interpretation bias in social situations • Use experimental paradigms to look at manipulation of focus of attention in social interaction task, paying attention to how switching between internal and external focus of attention may be influenced by executive function differences such as cognitive flexibility, interoceptive sensibility, and sensory differences of both internal and external sensory cues
H3. Reduced processing of external social cues when anxious	RQ1 S1 RQ2 S4	• Use experimental paradigms such as look at differences in processing time for face versus non-social stimuli under conditions that elicit high versus low SA in autistic and non-autistic individuals matched on SA • Compare recall of internal versus external information following social and non-social interactions in autistic individuals with high vs. low levels of co-occurring SA
H4. Generate distorted observer-perspective images of how they think they appear to others when in feared social situations	RQ1 S2 RQ2 S3	• Use cognitive interview approach to explore how autistic individuals are interpreting questionnaires that look at fear of negative evaluation and self-imagery in relation to their autistic identity and SA, to ensure that the questionnaire has good construct validity in assessing SA related cognitions • Explore quality of self-imagery generated by autistic individuals based on internal bodily cues and internal focus of attention, and how such imagery may influence maintenance of SA in autistic individuals
H5. Use internal information made accessible by self-focussed attention to make (erroneous) inferences about how they appear to others	RQ1 S3 RQ2 S6	• Explore how alexithymia and sensory processing differences moderate the relationship between emotion regulation difficulties endorsed by autistic young adults and self-/caregiver-reported SA • Explore the relationship between somatic and cognitive symptoms of anxiety and processing of self as a social object in autistic individuals • Explore how perceived social danger may be associated with changes in somatic and cognitive symptoms of SA in autistic individuals, how such symptoms may be used to inform processing of self in social situations, and whether associations are moderated by emotion regulation strategies
H6. In situation safety behaviour and self-focussed attention prevent disconfirmation of negative beliefs and maintain social phobia	RQ1 S4 RQ2 S7	• Explore differences in motivations for adopting behaviours that look like safety behaviours from SA literature, for example, camouflaging and hiding autistic traits to try to fit in with non-autistic individuals may provide an alternative rationale for engaging in masking behaviours analogous to impression management behaviours
H7. In situation safety behaviours and self-focussed attention can contaminate social interactions by making them less appealing to others	RQ1 S4 RQ2 S7	• Explore autistic individuals’ self-perceptions and associated beliefs about the role of different safety behaviours related to SA, and camouflaging behaviours related to masking autistic traits • Explore how dropping safety behaviours (associated with social anxiety) and camouflaging behaviours (associated with masking autistic traits) may differentially influence observer ratings of social performance on tasks that evoke high versus low levels of SA in autistic individuals • Examine the differential effects on social anxiety related cognitions, anxious feelings, and ratings by others of safety behaviours (both impression management and avoidance) and camouflaging
H8. Reduced processing of external social cues is biased in favour of detection and recall of cues that could be interpreted as signs of disapproval from others	RQ2 S4	• Use experimental paradigms to look at autistic individuals’ ability to detect and recall positive and negative audience behaviours during and after a performance-based task that evokes high levels of SA. Explore whether ability is associated with co-occurring SA symptoms. A matched non-autistic group with similar levels of SA can address whether differences in detection/recall of positive versus negative audience behaviours is related to SA or potential social communication differences associated with autistic traits
H9. Engage in negatively biased anticipatory processing before entering feared social situations	None to date	• Use experimental paradigms to explore recall of positive and negative words associated with others’ perception of self, one’s own perception of self, versus perception of others, following performance task that evokes high SA (e.g. giving a talk) versus distraction task • Qualitative studies to explore anticipatory processes autistic individuals engage in prior to social situations, paying close attention to potential overlap with camouflaging/masking behaviours • Explore how anticipatory behaviours affect one’s performance in tasks that evoke high versus low levels of SA
H10. Engage in prolonged, negatively biased, post-event processing	None to date	• Explore recall of positive and negative self-related information after performance tasks that involve stressful social interactions or evoke high levels of SA • Qualitative interviews to explore content and nature of imagery associated with post-event processing after stressful social situations. Pay close attention to the function of behaviour and specificity to SA, as post-event processing may be related gaining understanding of social situation (i.e. as a compensating behaviour for social communication differences), or related to co-occurring conditions such as depression/generalised anxiety

## Clinical Implications

Similar to recommendations outlined by Wilson and Gullon-Scott (2023), given the mixed evidence available, clinicians working with autistic individuals to target SA can benefit from carefully reviewing the cognitive model of social anxiety and discuss any questionnaire-based assessment outcomes with the client to understand how they made sense of the questions, drawing on their lived experiences as an autistic individual. We outline some suggested questions to support assessment and formulation of maintenance factors of social anxiety in autistic individuals in Fig. 2b. In contrast to the original Clark and Wells (1995) model which assumed individuals with SA do not have underlying social skills deficits, clinicians working with autistic individuals can guide person-centred conversations that centres on the autistic person’s lived experiences and ask autistic clients about how they may have modified their behaviours in social situations as a result of feedback they may have received from social partners in the past. Discussions of the autistic individual’s sense of autistic identity may also provide insight into potential internalised stigma that can contribute towards negative self-imagery in social situations, and distinctions between the importance of being autistic to one’s identity (i.e. autism centrality) and autism satisfaction may also help clinicians understand how autistic identity may activate assumptions and beliefs about oneself in social situations (Cooper et al., 2022a, 2022b; Davies et al., 2023). Acknowledging that clients may experience distress when recalling difficult social experiences, clinicians need to approach conversations with sensitivity and provide validation when listening to autistic people’s lived experiences.

Given construct overlap between camouflaging and safety behaviours (Lei et al., 2023; Wilson & Gullon-Scott, 2023), clinicians need to pay attention to the distinction the client may have made between camouflaging and safety behaviours, and specifically enquire whether the client is engaging in either type of behaviours in social situations. Clinicians can collaborate with autistic clients to carefully plan behavioural experiments to explore which behaviours they are willing to change during the experiment, distinguishing between safety behaviours and camouflaging behaviours where possible. Validating the autistic clients’ potential lived experience of negative social interactions related to social communication differences is crucial, and clinicians and autistic clients may benefit from considering the possibility of potential negative appraisal from others if social skills difficulties are evident, risk assess for potential victimisation or negative social experiences in the specific social situation and make a cope ahead plan if necessary. Finally, clinicians should explore ways of scaffolding social support within the autistic individual’s environment (e.g. peer mentoring, coaching, self-advocacy work) that may positively influence on the autistic individual’s perception of self and connection with one’s autistic identity (Davies et al., 2023).

## Supplementary Information

Below is the link to the electronic supplementary material.

Supplementary file1 (DOCX 32 KB)

## Data Availability

Template data collection forms, data extracted from included studies, and data used for all analyses can be made available upon request by contacting the corresponding author.

## Footnotes

1 Prominent processes that maintain social anxiety as outlined in the Wong and Rapee (2016) review are shown in brackets to highlight specific maintenance factors.

## References

[CR1] Albano, A. M., Marten, P. A., Holt, C. S., Heimberg, R. G., & Barlow, D. H. (1995). Cognitive-behavioral group treatment for social phobia in adolescents a preliminary study. *The Journal of Nervous and Mental Disease,**183*(10), 649. 7561811 10.1097/00005053-199510000-00006

[CR2] Albantakis, L., Brandi, M.-L., Zillekens, I. C., Henco, L., Weindel, L., Thaler, H., Schliephake, L., Timmermans, B., & Schilbach, L. (2020). Alexithymic and autistic traits: Relevance for comorbid depression and social phobia in adults with and without autism spectrum disorder. *Autism,**24*(8), 2046–2056. 10.1177/1362361320936024 32662285 10.1177/1362361320936024 PMC7543015

[CR3] Ambler, P. G., Eidels, A., & Gregory, C. (2015). Anxiety and aggression in adolescents with autism spectrum disorders attending mainstream schools. *Research in Autism Spectrum Disorders,**18*, 97–109. 10.1016/j.rasd.2015.07.005

[CR4] Antezana, L., Valdespino, A., Wieckowski, A. T., Coffman, M. C., Carlton, C. N., Garcia, K. M., Gracanin, D., White, S. W., & Richey, J. A. (2023). Social anxiety symptoms predict poorer facial emotion recognition in autistic male adolescents and young adults without intellectual disability. *Journal of Autism and Developmental Disorders*. 10.1007/s10803-023-05998-5 37120659 10.1007/s10803-023-05998-5 PMC12922774

[CR5] Asher, M., Asnaani, A., & Aderka, I. M. (2017). Gender differences in social anxiety disorder: A review. *Clinical Psychology Review,**56*, 1–12. 10.1016/j.cpr.2017.05.004 28578248 10.1016/j.cpr.2017.05.004

[CR6] Bagby, R. M., Parker, J. D. A., & Taylor, G. J. (1994). The twenty-item Toronto Alexithymia scale—I. Item selection and cross-validation of the factor structure. *Journal of Psychosomatic Research,**38*(1), 23–32. 10.1016/0022-3999(94)90005-1 8126686 10.1016/0022-3999(94)90005-1

[CR7] Barrett, P. M., Rapee, R. M., Dadds, M. M., & Ryan, S. M. (1996). Family enhancement of cognitive style in anxious and aggressive children. *Journal of Abnormal Child Psychology,**24*(2), 187–203. 10.1007/BF01441484 8743244 10.1007/BF01441484

[CR8] Barry, T. D., Klinger, L. G., Lee, J. M., Palardy, N., Gilmore, T., & Bodin, S. D. (2003). Examining the effectiveness of an outpatient clinic-based social skills group for high-functioning children with autism. *Journal of Autism and Developmental Disorders,**33*(6), 685–701. 10.1023/b:jadd.0000006004.86556.e0 14714936 10.1023/b:jadd.0000006004.86556.e0

[CR9] Beidel, D. C., Turner, S. M., & Morris, T. L. (1999). Psychopathology of childhood social phobia. *Journal of the American Academy of Child and Adolescent Psychiatry,**38*(6), 643–650. 10.1097/00004583-199906000-00010 10361781 10.1097/00004583-199906000-00010

[CR10] Bellini, S. (2004). Social skill deficits and anxiety in high-functioning adolescents with autism spectrum disorders. *Focus on Autism and Other Developmental Disabilities,**19*(2), 78–86. 10.1177/10883576040190020201

[CR11] Bellini, S. (2006). The development of social anxiety in adolescents with autism spectrum disorders. *Focus on Autism and Other Developmental Disabilities,**21*(3), 138–145. 10.1177/10883576060210030201

[CR12] Black, K. R., Stevenson, R. A., Segers, M., Ncube, B. L., Sun, S. Z., Philipp-Muller, A., Bebko, J. M., Barense, M. D., & Ferber, S. (2017). Linking anxiety and insistence on sameness in autistic children: the role of sensory hypersensitivity. *Journal of Autism and Developmental Disorders,**47*(8), 2459–2470. 10.1007/s10803-017-3161-x 28540453 10.1007/s10803-017-3161-x

[CR13] Blakeley-Smith, A., Reaven, J., Ridge, K., & Hepburn, S. (2012). Parent–child agreement of anxiety symptoms in youth with autism spectrum disorders. *Research in Autism Spectrum Disorders,**6*(2), 707–716. 10.1016/j.rasd.2011.07.020

[CR14] Blakemore, S.-J., & Robbins, T. W. (2012). Decision-making in the adolescent brain. *Nature Neuroscience,**15*(9), 1184–1191. 10.1038/nn.3177 22929913 10.1038/nn.3177

[CR15] Boelen, P. A., Vrinssen, I., & van Tulder, F. (2010). Intolerance of uncertainty in adolescents: Correlations with worry, social anxiety, and depression. *Journal of Nervous and Mental Disease,**198*(3), 194–200. 10.1097/NMD.0b013e3181d143de 20215996 10.1097/NMD.0b013e3181d143de

[CR16] Boulter, C., Freeston, M., South, M., & Rodgers, J. (2014). Intolerance of uncertainty as a framework for understanding anxiety in children and adolescents with autism spectrum disorders. *Journal of Autism and Developmental Disorders,**44*(6), 1391–1402. 10.1007/s10803-013-2001-x 24272526 10.1007/s10803-013-2001-x

[CR17] Boulton, K. A., & Guastella, A. J. (2021). Social anxiety symptoms in autism spectrum disorder and social anxiety disorder: Considering the reliability of self-report instruments in adult cohorts. *Autism Research: Official Journal of the International Society for Autism Research,**14*(11), 2383–2392. 10.1002/aur.2572 34213050 10.1002/aur.2572

[CR18] Campbell, M., McKenzie, J. E., Sowden, A., Katikireddi, S. V., Brennan, S. E., Ellis, S., Hartmann-Boyce, J., Ryan, R., Shepperd, S., Thomas, J., Welch, V., & Thomson, H. (2020). Synthesis without meta-analysis (SWiM) in systematic reviews: Reporting guideline. *BMJ,**368*, l6890. 10.1136/bmj.l6890 31948937 10.1136/bmj.l6890 PMC7190266

[CR19] Cardaciotto, L., & Herbert, J. D. (2004). Cognitive behavior therapy for social anxiety disorder in the context of Asperger’s Syndrome: A single-subject report. *Cognitive and Behavioral Practice,**11*(1), 75–81. 10.1016/S1077-7229(04)80009-9

[CR20] Chang, Y.-C., Quan, J., & Wood, J. J. (2012). Effects of Anxiety Disorder Severity on Social Functioning in Children with Autism Spectrum Disorders. *Journal of Developmental and Physical Disabilities,**24*(3), 235–245. 10.1007/s10882-012-9268-2

[CR21] Clark, D. M. (2003). *Social anxiety process measures*. Unpublished.

[CR22] Clark, D. M. (2005). Chapter 9: A cognitive perspective on social phobia. In R. Crozier & L. E. Alden (Eds.), *The essential handbook of social anxiety for clinicians. *John Wiley & Sons Ltd.

[CR23] Clark, D. M., Ehlers, A., Hackmann, A., McManus, F., Fennell, M., Grey, N., Waddington, L., & Wild, J. (2006). Cognitive therapy versus exposure and applied relaxation in social phobia: A randomized controlled trial. *Journal of Consulting and Clinical Psychology,**74*(3), 568–578. 10.1037/0022-006X.74.3.568 16822113 10.1037/0022-006X.74.3.568

[CR24] Clark, D. M., Ehlers, A., McManus, F., Hackmann, A., Fennell, M., Campbell, H., Flower, T., Davenport, C., & Louis, B. (2003). Cognitive therapy versus fluoxetine in generalized social phobia: A randomized placebo-controlled trial. *Journal of Consulting and Clinical Psychology,**71*(6), 1058–1067. 10.1037/0022-006X.71.6.1058 14622081 10.1037/0022-006X.71.6.1058

[CR25] Clark, D. M., & Wells, A. (1995). A cognitive model of social phobia. In R. G. Heimberg, M. R. Liebowitz, D. A. Hope, & F. R. Schneier (Eds.), *Social phobia: Diagnosis, assessment, and treatment* (pp. 69–93). The Guilford Press.

[CR26] Cohen, J. (1960). A coefficient of agreement for nominal scales. *Educational and Psychological Measurement,**20*, 37–46. 10.1177/001316446002000104

[CR27] Cohen, T. R., Wolf, S. T., Panter, A. T., & Insko, C. A. (2011). Introducing the GASP scale: A new measure of guilt and shame proneness. *Journal of Personality and Social Psychology,**100*(5), 947–966. 10.1037/a0022641 21517196 10.1037/a0022641

[CR28] Cook, J., Hull, L., Crane, L., & Mandy, W. (2021). Camouflaging in autism: A systematic review. *Clinical Psychology Review,**89*, 102080. 10.1016/j.cpr.2021.102080 34563942 10.1016/j.cpr.2021.102080

[CR29] Cooper, K., Mandy, W., Butler, C., & Russell, A. (2022a). The lived experience of gender dysphoria in autistic adults: An interpretative phenomenological analysis. *Autism,**26*(4), 963–974. 10.1177/13623613211039113 34376079 10.1177/13623613211039113 PMC9014767

[CR30] Cooper, K., Russell, A., Lei, J., & Smith, L. G. E. (2022b). The impact of a positive autism identity and autistic community solidarity on social anxiety and mental health in autistic young people. *Autism*. 10.1177/13623613221118351 36062470 10.1177/13623613221118351 PMC10074754

[CR31] Corden, B., Chilvers, R., & Skuse, D. (2008). Avoidance of emotionally arousing stimuli predicts social-perceptual impairment in Asperger’s syndrome. *Neuropsychologia,**46*(1), 137–147. 10.1016/j.neuropsychologia.2007.08.005 17920642 10.1016/j.neuropsychologia.2007.08.005

[CR32] Davies, J., Cooper, K., Killick, E., Sam, E., Healy, M., Thompson, G., Mandy, W., Redmayne, B., & Crane, L. (2023). Autistic identity: A systematic review of quantitative research. *OSF Preprints*. 10.31219/osf.io/74k6m 10.1002/aur.3105 38334318

[CR33] Deckers, A., Muris, P., & Roelofs, J. (2017). Being on your own or feeling lonely? Loneliness and other social variables in youths with autism spectrum disorders. *Child Psychiatry & Human Development,**48*(5), 828–839. 10.1007/s10578-016-0707-7 28070762 10.1007/s10578-016-0707-7 PMC5617879

[CR34] Deckers, A., Muris, P., Roelofs, J., & Arntz, A. (2016). A Group-administered social skills training for 8- to 12- year-old, high-functioning children with autism spectrum disorders: An evaluation of its effectiveness in a naturalistic outpatient treatment setting. *Journal of Autism and Developmental Disorders,**46*(11), 3493–3504. 10.1007/s10803-016-2887-1 27522220 10.1007/s10803-016-2887-1 PMC5073106

[CR35] Dekkers, O. M., Vandenbroucke, J. P., Cevallos, M., Renehan, A. G., Altman, D. G., & Egger, M. (2019). COSMOS-E: Guidance on conducting systematic reviews and meta-analyses of observational studies of etiology. *PLoS Medicine*. 10.1371/journal.pmed.1002742 30789892 10.1371/journal.pmed.1002742 PMC6383865

[CR36] Demetriou, E. A., Lampit, A., Quintana, D. S., Naismith, S. L., Song, Y. J. C., Pye, J. E., Hickie, I., & Guastella, A. J. (2018). Autism spectrum disorders: A meta-analysis of executive function. *Molecular Psychiatry,**23*(5), 1198–1204. 10.1038/mp.2017.75 28439105 10.1038/mp.2017.75 PMC5984099

[CR37] Demetriou, E. A., Park, S. H., Ho, N., Pepper, K. L., Song, Y. J. C., Naismith, S. L., Thomas, E. E., Hickie, I. B., & Guastella, A. J. (2020). Machine learning for differential diagnosis between clinical conditions with social difficulty: Autism spectrum disorder, early psychosis, and social anxiety disorder. *Frontiers in Psychiatry*. 10.3389/fpsyt.2020.00545 32636768 10.3389/fpsyt.2020.00545 PMC7319094

[CR38] Detweiler, M. F., Comer, J. S., Crum, K. I., & Albano, A. M. (2014). Chapter 10 - Social Anxiety in Children and Adolescents: Biological, Developmental, and Social Considerations. In S. G. Hofmann & P. M. DiBartolo (Eds.), *Social Anxiety* (3rd ed., pp. 253–309). Academic Press.

[CR39] Dugas, M. J. (2004). *CBT for GAD: Learning to tolerate uncertainty and emotional arousal. Manual to accompnay workshop at 34th European Association for Behavioural and Cognitive Therapies (EABCT) Conference*.

[CR40] Espelöer, J., Hellmich, M., Vogeley, K., & Falter-Wagner, C. M. (2021). Brief report: Social anxiety in autism spectrum disorder is based on deficits in social competence. *Journal of Autism and Developmental Disorders,**51*(1), 315–322. 10.1007/s10803-020-04529-w 32410100 10.1007/s10803-020-04529-w PMC7810630

[CR41] Fombonne, E. (2020). Camouflage and autism. *Journal of Child Psychology and Psychiatry,**61*(7), 735–738. 10.1111/jcpp.13296 32658354 10.1111/jcpp.13296

[CR42] Gadow, K. D., & Garman, H. D. (2020). Social anhedonia in children and adolescents with autism spectrum disorder and psychiatry referrals. *Journal of Clinical Child & Adolescent Psychology,**49*(2), 239–250. 10.1080/15374416.2018.1514611 30412420 10.1080/15374416.2018.1514611

[CR43] Gaziel-Guttman, M., Anaki, D., & Mashal, N. (2023). Social anxiety and shame among young adults with autism spectrum disorder compared to typical adults. *Journal of Autism and Developmental Disorders,**53*(6), 2490–2498. 10.1007/s10803-022-05526-x 35394242 10.1007/s10803-022-05526-x

[CR44] Gratz, K. L., & Roemer, L. (2004). Multidimensional assessment of emotion regulation and dysregulation: development, factor structure, and initial validation of the difficulties in emotion regulation scale. *Journal of Psychopathology and Behavioral Assessment,**26*(1), 41–54. 10.1023/B:JOBA.0000007455.08539.94

[CR45] Gray, S. A. O., & Carter, A. S. (2013). Adaptive behavior assessment system, second edition. In F. R. Volkmar (Ed.), *Encyclopedia of autism spectrum disorders* (pp. 52–55). Springer.

[CR46] Greenberg, C. I., Strube, M. J., & Myers, R. A. (1980). A multitrait-multimethod investigation of interpersonal distance. *Journal of Nonverbal Behavior,**5*(2), 104–114. 10.1007/BF00986513

[CR47] Gresham, F., & Elliott, S. N. (2008). *Social skills improvement system (SSIS) rating scale*. Pearson Assessment.

[CR48] Halldorsson, B., & Creswell, C. (2017). Social anxiety in pre-adolescent children: What do we know about maintenance? *Behaviour Research and Therapy,**99*, 19–36. 10.1016/j.brat.2017.08.013 28881221 10.1016/j.brat.2017.08.013

[CR49] Hedley, D., & Young, R. (2006). Social comparison processes and depressive symptoms in children and adolescents with Asperger syndrome. *Autism,**10*(2), 139–153. 10.1177/1362361306062020 16613864 10.1177/1362361306062020

[CR50] Hollocks, M. J., Leno, V. C., Chandler, S., White, P., Yorke, I., Charman, T., Pickles, A., Baird, G., & Simonoff, E. (2022). Psychiatric conditions in autistic adolescents: Longitudinal stability from childhood and associated risk factors. *European Child & Adolescent Psychiatry*. 10.1007/s00787-022-02065-9 10.1007/s00787-022-02065-9 PMC10576662 35976471

[CR51] Hollocks, M. J., Lerh, J. W., Magiati, I., Meiser-Stedman, R., & Brugha, T. S. (2019). Anxiety and depression in adults with autism spectrum disorder: A systematic review and meta-analysis. *Psychological Medicine,**49*(4), 559–572. 10.1017/S0033291718002283 30178724 10.1017/S0033291718002283

[CR52] Hollocks, M. J., Ozsivadjian, A., Matthews, C. E., Howlin, P., & Simonoff, E. (2013). The relationship between attentional bias and anxiety in children and adolescents with autism spectrum disorders. *Autism Research: Official Journal of the International Society for Autism Research,**6*(4), 237–247. 10.1002/aur.1285 23907924 10.1002/aur.1285

[CR53] Hollocks, M. J., Pickles, A., Howlin, P., & Simonoff, E. (2016). Dual cognitive and biological correlates of anxiety in autism spectrum disorders. *Journal of Autism and Developmental Disorders,**46*(10), 3295–3307. 10.1007/s10803-016-2878-2 27465243 10.1007/s10803-016-2878-2

[CR54] Hull, L., Levy, L., Lai, M.-C., Petrides, K. V., Baron-Cohen, S., Allison, C., Smith, P., & Mandy, W. (2021). Is social camouflaging associated with anxiety and depression in autistic adults? *Molecular Autism,**12*(1), 13. 10.1186/s13229-021-00421-1 33593423 10.1186/s13229-021-00421-1 PMC7885456

[CR55] Hull, L., Mandy, W., Lai, M.-C., Baron-Cohen, S., Allison, C., Smith, P., & Petrides, K. V. (2019). Development and validation of the camouflaging autistic traits questionnaire (CAT-Q). *Journal of Autism and Developmental Disorders,**49*(3), 819–833. 10.1007/s10803-018-3792-6 30361940 10.1007/s10803-018-3792-6 PMC6394586

[CR56] Hull, L., Petrides, K. V., Allison, C., Smith, P., Baron-Cohen, S., Lai, M.-C., & Mandy, W. (2017). “Putting on my best normal”: Social camouflaging in adults with autism spectrum conditions. *Journal of Autism and Developmental Disorders,**47*(8), 2519–2534. 10.1007/s10803-017-3166-5 28527095 10.1007/s10803-017-3166-5 PMC5509825

[CR57] Ingul, J. M., Aune, T., & Nordahl, H. M. (2014). A randomized controlled trial of individual cognitive therapy, group cognitive behaviour therapy and attentional placebo for adolescent social phobia. *Psychotherapy and Psychosomatics,**83*(1), 54–61. 10.1159/000354672 24281563 10.1159/000354672

[CR58] John, T., Woods, S., Bode, T., Ritter, C., & Estes, A. (2022). A review of executive functioning challenges and strengths in autistic adults. *The Clinical Neuropsychologist,**36*(5), 1116–1147. 10.1080/13854046.2021.1971767 34499568 10.1080/13854046.2021.1971767

[CR59] Johnson, H. S., Inderbitzen-Nolan, H. M., & Anderson, E. R. (2006). The Social Phobia Inventory: Validity and reliability in an adolescent community sample. *Psychological Assessment,**18*(3), 269–277. 10.1037/1040-3590.18.3.269 16953730 10.1037/1040-3590.18.3.269

[CR60] Kaboski, J. R., Diehl, J. J., Beriont, J., Crowell, C. R., Villano, M., Wier, K., & Tang, K. (2015). Brief report: A pilot summer robotics camp to reduce social anxiety and improve social/vocational skills in adolescents with ASD. *Journal of Autism and Developmental Disorders,**45*(12), 3862–3869. 10.1007/s10803-014-2153-3 24898910 10.1007/s10803-014-2153-3

[CR61] Kanat, M., Spenthof, I., Riedel, A., van Elst, L. T., Heinrichs, M., & Domes, G. (2017). Restoring effects of oxytocin on the attentional preference for faces in autism. *Translational Psychiatry,**7*(4), e1097. 10.1038/tp.2017.67 28418399 10.1038/tp.2017.67 PMC5416705

[CR62] Kang, E., Clarkson, T., Keifer, C. M., Rosen, T. E., & Lerner, M. D. (2019). Discrete electrocortical predictors of anxiety and anxiety-related treatment response in youth with autism spectrum disorder. *Biological Psychology,**146*, 107710. 10.1016/j.biopsycho.2019.05.010 31158425 10.1016/j.biopsycho.2019.05.010

[CR63] Kazdin, A. E. (2007). Mediators and mechanisms of change in psychotherapy research. *Annual Review of Clinical Psychology,**3*, 1–27. 10.1146/annurev.clinpsy.3.022806.091432 17716046 10.1146/annurev.clinpsy.3.022806.091432

[CR64] Kessler, R. C., Berglund, P., Demler, O., Jin, R., Merikangas, K. R., & Walters, E. E. (2005). Lifetime prevalence and age-of-onset distributions of DSM-IV disorders in the National Comorbidity Survey Replication. *Archives of General Psychiatry,**62*(6), 593–602. 10.1001/archpsyc.62.6.593 15939837 10.1001/archpsyc.62.6.593

[CR65] Kimura, Y., Fujioka, T., Jung, M., Fujisawa, T. X., Tomoda, A., & Kosaka, H. (2020). An investigation of the effect of social reciprocity, social anxiety, and letter fluency on communicative behaviors in adults with autism spectrum disorder. *Psychiatry Research,**294*, 113503. 10.1016/j.psychres.2020.113503 33113450 10.1016/j.psychres.2020.113503

[CR66] Kmet, L., Lee, R. C., Cook, L. S., Lorenzetti, D., Godlovitch, G., & Einsiedel, E. (2004). *Standard quality assessment cirteria for evaluating primary research papers from a variety of fields*. Alberta Heritage Foundatoin for Medical Research.

[CR67] Kuusikko, S., Pollock-Wurman, R., Jussila, K., Carter, A. S., Mattila, M.-L., Ebeling, H., Pauls, D. L., & Moilanen, I. (2008). Social anxiety in high-functioning children and adolescents with autism and asperger syndrome. *Journal of Autism and Developmental Disorders,**38*(9), 1697–1709. 10.1007/s10803-008-0555-9 18324461 10.1007/s10803-008-0555-9

[CR68] La Greca, A. M., Ingles, C. J., Lai, B. S., & Marzo, J. C. (2015). Social anxiety scale for adolescents: Factorial invariance across gender and age in hispanic american adolescents. *Assessment,**22*(2), 224–232. 10.1177/1073191114540749 25059683 10.1177/1073191114540749

[CR69] Lei, J., Charman, T., Leigh, E., Russell, A., Mohamed, Z., & Hollocks, M. J. (2022). Examining the relationship between cognitive inflexibility and internalizing and externalizing symptoms in autistic children and adolescents: A systematic review and meta-analysis. *Autism Research,**15*(12), 2265–2295. 10.1002/aur.2826 36196666 10.1002/aur.2826 PMC10092776

[CR70] Lei, J., Leigh, E., Charman, T., Russell, A., & Hollocks, M. J. (2023). Understanding the relationship between social camouflaging in autism and safety behaviours in social anxiety in autistic and non-autistic adolescents. *Journal of Child Psychology and Psychiatry*. 10.1111/jcpp.13884 37632264 10.1111/jcpp.13884

[CR71] Leigh, E., & Clark, D. M. (2018). Understanding social anxiety disorder in adolescents and improving treatment outcomes: applying the cognitive model of Clark and Wells (1995). *Clinical Child and Family Psychology Review,**21*(3), 388–414. 10.1007/s10567-018-0258-5 29654442 10.1007/s10567-018-0258-5 PMC6447508

[CR72] Leigh, E., & Clark, D. M. (2023). Internet-delivered therapist-assisted cognitive therapy for adolescent social anxiety disorder (OSCA): A randomised controlled trial addressing preliminary efficacy and mechanisms of action. *Journal of Child Psychology and Psychiatry,**64*(1), 145–155. 10.1111/jcpp.13680 35943064 10.1111/jcpp.13680 PMC10087225

[CR73] Lerner, M. D., White, S. W., & McPartland, J. C. (2012). Mechanisms of change in psychosocial interventions for autism spectrum disorders. *Dialogues in Clinical Neuroscience,**14*(3), 307–318. 10.31887/DCNS.2012.14.3/mlerner 23226955 10.31887/DCNS.2012.14.3/mlerner PMC3513684

[CR74] Liebowitz, M. R. (1987). Social phobia. *Modern Problems of Pharmacopsychiatry,**22*, 141–173. 10.1159/000414022 2885745 10.1159/000414022

[CR75] Ludlow, A. K., Osborne, C., & Keville, S. (2023). Selective mutism in children with and without an autism spectrum disorder: The role of sensory avoidance in mediating symptoms of social anxiety. *Journal of Autism and Developmental Disorders,**53*(10), 3891–3900. 10.1007/s10803-022-05674-0 35904647 10.1007/s10803-022-05674-0

[CR76] Luxford, S., Hadwin, J. A., & Kovshoff, H. (2017). Evaluating the effectiveness of a school-based cognitive behavioural therapy intervention for anxiety in adolescents diagnosed with autism spectrum disorder. *Journal of Autism and Developmental Disorders,**47*(12), 3896–3908. 10.1007/s10803-016-2857-7 27440250 10.1007/s10803-016-2857-7 PMC5676836

[CR77] MacLennan, K., Roach, L., & Tavassoli, T. (2020). The relationship between sensory reactivity differences and anxiety subtypes in autistic children. *Autism Research: Official Journal of the International Society for Autism Research,**13*(5), 785–795. 10.1002/aur.2259 31909874 10.1002/aur.2259

[CR78] Maddox, B. B., & White, S. W. (2015). Comorbid social anxiety disorder in adults with autism spectrum disorder. *Journal of Autism and Developmental Disorders,**45*(12), 3949–3960. 10.1007/s10803-015-2531-5 26243138 10.1007/s10803-015-2531-5

[CR79] Mahon, C. P., Pachankis, J. E., Kiernan, G., & Gallagher, P. (2023). Social anxiety across sexual and gender identity subpopulations. *Annals of LGBTQ Public and Population Health,**4*(1), 26–41. 10.1891/LGBTQ-2020-0017

[CR80] May, T., Cornish, K., & Rinehart, N. J. (2015). Mechanisms of anxiety related attentional biases in children with autism spectrum disorder. *Journal of Autism and Developmental Disorders,**45*(10), 3339–3350. 10.1007/s10803-015-2500-z 26070278 10.1007/s10803-015-2500-z

[CR81] Mayo-Wilson, E., Dias, S., Mavranezouli, I., Kew, K., Clark, D. M., Ades, A. E., & Pilling, S. (2014). Psychological and pharmacological interventions for social anxiety disorder in adults: A systematic review and network meta-analysis. *The Lancet Psychiatry,**1*(5), 368–376. 10.1016/S2215-0366(14)70329-3 26361000 10.1016/S2215-0366(14)70329-3 PMC4287862

[CR82] McHugh, M. L. (2012). Interrater reliability: The kappa statistic. *Biochemia Medica,**22*(3), 276–282. 23092060 PMC3900052

[CR83] Mesa, F., Le, T.-A., & Beidel, D. C. (2015). Social skill-based treatment for social anxiety disorder in adolescents. In K. Ranta, A. M. La Greca, L.-J. Garcia-Lopez, & M. Marttunen (Eds.), *Social anxiety and phobia in adolescents: Development, manifestation and intervention strategies* (pp. 289–299). Springer International Publishing.

[CR84] Mörtberg, E., Clark, D. M., Sundin, Ö., & Åberg Wistedt, A. (2007). Intensive group cognitive treatment and individual cognitive therapy vs. treatment as usual in social phobia: A randomized controlled trial. *Acta Psychiatrica Scandinavica,**115*(2), 142–154. 10.1111/j.1600-0447.2006.00839.x 17244178 10.1111/j.1600-0447.2006.00839.x

[CR85] National Institute for Health and Care Excellence. (2013). *1 Recommendations | Social anxiety disorder: Recognition, assessment and treatment | Guidance | NICE*. NICE. https://www.nice.org.uk/guidance/cg159/chapter/recommendations 31869048

[CR86] Norcross, J. C., & Wampold, B. E. (2011). What works for whom: Tailoring psychotherapy to the person. *Journal of Clinical Psychology,**67*(2), 127–132. 10.1002/jclp.20764 21108312 10.1002/jclp.20764

[CR87] Ozsivadjian, A., Hollocks, M. J., Magiati, I., Happé, F., Baird, G., & Absoud, M. (2021). Is cognitive inflexibility a missing link? The role of cognitive inflexibility, alexithymia and intolerance of uncertainty in externalising and internalising behaviours in young people with autism spectrum disorder. *Journal of Child Psychology and Psychiatry, and Allied Disciplines,**62*(6), 715–724. 10.1111/jcpp.13295 32827150 10.1111/jcpp.13295

[CR88] Page, M. J., McKenzie, J. E., Bossuyt, P. M., Boutron, I., Hoffmann, T. C., Mulrow, C. D., Shamseer, L., Tetzlaff, J. M., Akl, E. A., Brennan, S. E., Chou, R., Glanville, J., Grimshaw, J. M., Hróbjartsson, A., Lalu, M. M., Li, T., Loder, E. W., Mayo-Wilson, E., McDonald, S., & Moher, D. (2021). The PRISMA 2020 statement: An updated guideline for reporting systematic reviews. *Journal of Clinical Epidemiology,**134*, 178–189. 10.1016/j.jclinepi.2021.03.001 33789819 10.1016/j.jclinepi.2021.03.001

[CR89] Paul Wright, K. (2013). Cognitive behavioural therapy for anxiety in a man with autism spectrum disorder, intellectual disability, and social phobia. *Advances in Mental Health and Intellectual Disabilities,**7*(5), 284–292. 10.1108/AMHID-06-2013-0040

[CR90] Perry, A., Levy-Gigi, E., Richter-Levin, G., & Shamay-Tsoory, S. G. (2015). Interpersonal distance and social anxiety in autistic spectrum disorders: A behavioral and ERP study. *Social Neuroscience,**10*(4), 354–365. 10.1080/17470919.2015.1010740 25666260 10.1080/17470919.2015.1010740

[CR91] Pickard, H., Hirsch, C., Simonoff, E., & Happé, F. (2020). Exploring the cognitive, emotional and sensory correlates of social anxiety in autistic and neurotypical adolescents. *Journal of Child Psychology and Psychiatry*. 10.1111/jcpp.13214 32115711 10.1111/jcpp.13214 PMC7116440

[CR92] American Psychiatric Association. (2013). *Diagnostic and Statistical Manual of Mental Disorders (DSM-5®)*. American Psychiatric Pub.

[CR93] Rapee, R. M., & Heimberg, R. G. (1997). A cognitive-behavioral model of anxiety in social phobia. *Behaviour Research and Therapy,**35*(8), 741–756. 10.1016/S0005-7967(97)00022-3 9256517 10.1016/s0005-7967(97)00022-3

[CR94] Ringold, S. M., McGuire, R. W., Jayashankar, A., Kilroy, E., Butera, C. D., Harrison, L., Cermak, S. A., & Aziz-Zadeh, L. (2022). Sensory modulation in children with developmental coordination disorder compared to autism spectrum disorder and typically developing children. *Brain Sciences,**12*(9), 1171. 10.3390/brainsci12091171 36138908 10.3390/brainsci12091171 PMC9496992

[CR95] Russell, A., Gaunt, D. M., Cooper, K., Barton, S., Horwood, J., Kessler, D., Metcalfe, C., Ensum, I., Ingham, B., Parr, J. R., Rai, D., & Wiles, N. (2020). The feasibility of low-intensity psychological therapy for depression co-occurring with autism in adults: The autism depression trial (ADEPT) – A pilot randomised controlled trial. *Autism,**24*(6), 1360–1372. 10.1177/1362361319889272 31782656 10.1177/1362361319889272 PMC8645299

[CR96] Scharfstein, L. A., Beidel, D. C., Sims, V. K., & Rendon Finnell, L. (2011). Social skills deficits and vocal characteristics of children with social phobia or Asperger’s disorder: A comparative study. *Journal of Abnormal Child Psychology,**39*(6), 865–875. 10.1007/s10802-011-9498-2 21399935 10.1007/s10802-011-9498-2

[CR97] Schiltz, H. K., Magnus, B. E., McVey, A. J., Haendel, A. D., Dolan, B. K., Stanley, R. E., Willar, K. A., Pleiss, S. J., Carson, A. M., Carlson, M., Murphy, C., Vogt, E. M., Yund, B. D., & Van Hecke, A. V. (2019). A psychometric analysis of the social anxiety scale for adolescents among youth with autism spectrum disorder: Caregiver-adolescent agreement, factor structure, and validity. *Assessment*. 10.1177/1073191119851563 31165617 10.1177/1073191119851563 PMC9717689

[CR98] Schleismann, K. D., & Gillis, J. M. (2011). The Treatment of Social Phobia in a Young Boy With Asperger’s Disorder. *Cognitive and Behavioral Practice,**18*(4), 515–529. 10.1016/j.cbpra.2010.08.004

[CR99] Silverman, W., Albano, A., & Barlow, D. (1996). *Manual for the ADIS-IV-C/P*. Psychological Corporation.

[CR100] Simonoff, E., Pickles, A., Charman, T., Chandler, S., Loucas, T., & Baird, G. (2008). Psychiatric disorders in children with autism spectrum disorders: Prevalence, comorbidity, and associated factors in a population-derived sample. *Journal of the American Academy of Child & Adolescent Psychiatry,**47*(8), 921–929. 10.1097/CHI.0b013e318179964f 18645422 10.1097/CHI.0b013e318179964f

[CR101] Song, Y., Nie, T., Shi, W., Zhao, X., & Yang, Y. (2019). Empathy impairment in individuals with autism spectrum conditions from a multidimensional perspective: A meta-analysis. *Frontiers in Psychology*. https://www.frontiersin.org/journals/psychology/articles/10.3389/fpsyg.2019.01902 10.3389/fpsyg.2019.01902 PMC6794557 31649570

[CR102] Spain, D., Happé, F., Johnston, P., Campbell, M., Sin, J., Daly, E., Ecker, C., Anson, M., Chaplin, E., Glaser, K., Mendez, A., Lovell, K., & Murphy, D. G. (2016). Social anxiety in adult males with autism spectrum disorders. *Research in Autism Spectrum Disorders,**32*, 13–23. 10.1016/j.rasd.2016.08.002

[CR103] Spain, D., Sin, J., Harwood, L., Mendez, M. A., & Happé, F. (2017). Cognitive behaviour therapy for social anxiety in autism spectrum disorder: A systematic review. *Advances in Autism,**3*(1), 34–46. 10.1108/AIA-07-2016-0020

[CR104] Spain, D., Sin, J., Linder, K. B., McMahon, J., & Happé, F. (2018). Social anxiety in autism spectrum disorder: A systematic review. *Research in Autism Spectrum Disorders,**52*, 51–68. 10.1016/j.rasd.2018.04.007

[CR105] Stangier, U., Heidenreich, T., Peitz, M., Lauterbach, W., & Clark, D. M. (2003). Cognitive therapy for social phobia: Individual versus group treatment. *Behaviour Research and Therapy,**41*(9), 991–1007. 10.1016/S0005-7967(02)00176-6 12914803 10.1016/s0005-7967(02)00176-6

[CR106] Stangier, U., Schramm, E., Heidenreich, T., Berger, M., & Clark, D. M. (2011). Cognitive therapy vs interpersonal psychotherapy in social anxiety disorder: A randomized controlled trial. *Archives of General Psychiatry,**68*(7), 692–700. 10.1001/archgenpsychiatry.2011.67 21727253 10.1001/archgenpsychiatry.2011.67

[CR107] Stein, M. B., & Kean, Y. M. (2000). Disability and quality of life in social phobia: epidemiologic findings. *American Journal of Psychiatry,**157*(10), 1606–1613. 10.1176/appi.ajp.157.10.1606 11007714 10.1176/appi.ajp.157.10.1606

[CR108] Storch, E. A., Ehrenreich May, J., Wood, J. J., Jones, A. M., De Nadai, A. S., Lewin, A. B., Arnold, E. B., & Murphy, T. K. (2012). Multiple informant agreement on the anxiety disorders interview schedule in youth with autism spectrum disorders. *Journal of Child and Adolescent Psychopharmacology,**22*(4), 292–299. 10.1089/cap.2011.0114 22856332 10.1089/cap.2011.0114 PMC3422049

[CR109] Swain, D., Scarpa, A., White, S., & Laugeson, E. (2015). Emotion dysregulation and anxiety in adults with ASD: Does social motivation play a role? *Journal of Autism and Developmental Disorders,**45*(12), 3971–3977. 10.1007/s10803-015-2567-6 26319254 10.1007/s10803-015-2567-6

[CR110] Turner, M. A., & Hammond, N. (2016). Cognitive behavioural therapy in the treatment of social skills deficits and social phobia in a man with an autism spectrum disorder: A single-case study. *The Cognitive Behaviour Therapist,**9*, e3. 10.1017/S1754470X15000768

[CR111] Ung, D., McBride, N., Collier, A., Selles, R., Small, B., Phares, V., & Storch, E. (2016). The relationship between peer victimization and the psychological characteristics of youth with autism spectrum disorder. *Research in Autism Spectrum Disorders,**32*, 70–79. 10.1016/j.rasd.2016.09.002

[CR112] van der Miesen, A. I. R., Hurley, H., Bal, A. M., & de Vries, A. L. C. (2018). Prevalence of the wish to be of the opposite gender in adolescents and adults with autism spectrum disorder. *Archives of Sexual Behavior,**47*(8), 2307–2317. 10.1007/s10508-018-1218-3 29736809 10.1007/s10508-018-1218-3 PMC6245048

[CR113] van Schalkwyk, G., Smith, I. C., Silverman, W. K., & Volkmar, F. R. (2018). Brief report: bullying and anxiety in high-functioning adolescents with ASD. *Journal of Autism and Developmental Disorders,**48*(5), 1819–1824. 10.1007/s10803-017-3378-8 29152669 10.1007/s10803-017-3378-8

[CR114] White, S. W., Lerner, M. D., McLeod, B. D., Wood, J. J., Ginsburg, G. S., Kerns, C., Ollendick, T., Kendall, P. C., Piacentini, J., Walkup, J., & Compton, S. (2015). Anxiety in youth with and without autism spectrum disorder: Examination of factorial equivalence. *Behavior Therapy,**46*(1), 40–53. 10.1016/j.beth.2014.05.005 25526834 10.1016/j.beth.2014.05.005 PMC4273846

[CR115] Wilson, A. C., & Gullon-Scott, F. (2023). Social anxiety in autistic people: Does the Clark and Wells model fit? *Journal of Autism and Developmental Disorders*. 10.1007/s10803-023-06108-1 37751086 10.1007/s10803-023-06108-1 PMC11461584

[CR116] Wong, Q. J. J., & Rapee, R. M. (2016). The aetiology and maintenance of social anxiety disorder: A synthesis of complementary theoretical models and formulation of a new integrated model. *Journal of Affective Disorders,**203*, 84–100. 10.1016/j.jad.2016.05.069 27280967 10.1016/j.jad.2016.05.069

[CR117] Wood, H., Rusbridge, S., Lei, J., Lomax, C., Elliston, J., & Russell, A. (2022). Exploring the cognitive model of social anxiety in autistic young people—The central role of bodily symptoms. *Journal of Autism and Developmental Disorders,**52*(12), 5500–5514. 10.1007/s10803-021-05359-0 34865202 10.1007/s10803-021-05359-0 PMC9637062

[CR118] Wood, J. J., Kendall, P. C., Wood, K. S., Kerns, C. M., Seltzer, M., Small, B. J., Lewin, A. B., & Storch, E. A. (2020). Cognitive behavioral treatments for anxiety in children with autism spectrum disorder: A randomized clinical trial. *JAMA Psychiatry,**77*(5), 474–483. 10.1001/jamapsychiatry.2019.4160 31755906 10.1001/jamapsychiatry.2019.4160 PMC6902190

[CR119] Woody, S. R. (1996). Effects of focus of attention on anxiety levels and social performance of individuals with social phobia. *Journal of Abnormal Psychology,**105*(1), 61–69. 8666712 10.1037//0021-843x.105.1.61

[CR120] Woolard, A., Stratton, E., Demetriou, E. A., Boulton, K. A., Pellicano, E., Glozier, N., Gibbs, V., Rogerson, N., Quinn, P., Hickie, I. B., & Guastella, A. J. (2021). Perceptions of social and work functioning are related to social anxiety and executive function in autistic adults. *Autism: the International Journal of Research and Practice,**25*(7), 2124–2134. 10.1177/13623613211013664 34271838 10.1177/13623613211013664

[CR121] Yeung, M. K. (2022). A systematic review and meta-analysis of facial emotion recognition in autism spectrum disorder: The specificity of deficits and the role of task characteristics. *Neuroscience & Biobehavioral Reviews,**133*, 104518. 10.1016/j.neubiorev.2021.104518 34974069 10.1016/j.neubiorev.2021.104518

[CR122] Zukerman, G., Yahav, G., & Ben-Itzchak, E. (2020). The gap between cognition and adaptive behavior in students with autism spectrum disorder: Implications for social anxiety and the moderating effect of autism traits. *Journal of Autism and Developmental Disorders*. 10.1007/s10803-020-04632-y 32740852 10.1007/s10803-020-04632-y

